# Asymmetric Synthesis of Functionalized 2-Isoxazolines

**DOI:** 10.1021/acsomega.4c08062

**Published:** 2025-02-12

**Authors:** Beyza Hamur, Fatma Albayrak Halac, Fethiye Yilmazer, Fraser F. Fleming, Irem Kulu, Ramazan Altundas

**Affiliations:** † Department of Chemistry, College of Science, Gebze Technical University, 41400 Gebze, Kocaeli, Turkey; ‡ Department of Chemistry, Drexel University, 306 Disque Hall, Philadelphia, Pennsylvania 19104, United States

## Abstract

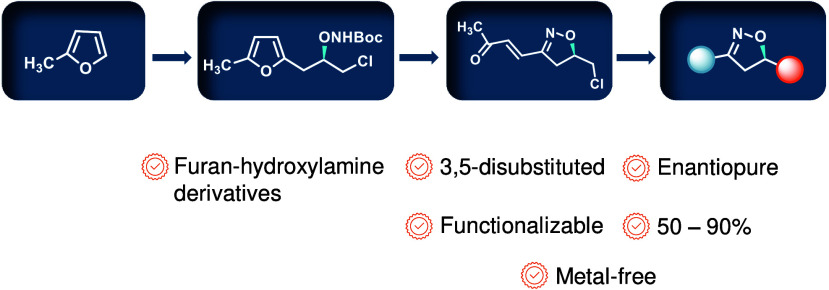

The asymmetric synthesis of the isoxazoline skeleton was achieved by the oxidation of hydroxylaminoalkyl furan prepared from simple starting materials: 2-methylfuran and (*S*)-epichlorohydrin. The synthesis features an NBS-mediated oxidation of hydroxylaminoalkyl furan to give an α,β-unsaturated ketone intermediate which cyclized to the isoxazoline. The α,β-unsaturated double bond was successfully cleaved with RuCl_3_·*x*H_2_O catalysis en route to the isoxazoline skeleton bearing alcohol, aldehyde, and carboxylic acid functionalities.

## Introduction

Isoxazolines are privileged heterocycles in medicinal and agrochemistry.^1−4^ They contain nitrogen and oxygen embedded in a five-membered cyclic skeleton, which are found in natural products, pharmaceuticals, and agrochemicals.^5−11^ Isoxazolines have attracted attention because of their versatility in the preparation of both cyclic and acyclic compounds and because of their diverse bioactivity. They exhibit a range of biological activities, including anti-inflammatory,^11^ anticancer,^6,12,13^ antifungal,^14^ and antibacterial profiles.^15−17^ In addition, isoxazolines are relatively common synthetic building blocks that provide access to amino alcohols, hydroxy ketones, isoxazolidines,^18−25^ and chiral ligands.^26^

Isoxazolines are often synthesized via [3 + 2] cycloaddition reactions between a nitrile oxide and alkene.^27^ The reaction can afford enantiomerically enriched isoxazolines by employing a chiral Lewis acid formed *in situ* from a pure allyl alcohol, generating chiral isoxazolines in high diastereoselectivity.^21,28^ Chiral ligand–metal complexes can also be used for the catalytic asymmetric syntheses of isoxazolines from nitrile oxides and alkenes, though control of the regioselectivity can be a significant challenge.^29−32^ Other valuable routes to isoxazoline include catalytic asymmetric [3 + 2] cycloaddition of a silyl nitronate with an alkene,^33−35^ enantioselective [4 + 1] annulation,^36^ cycloaddition of allylic oximes and hydroxylamine,^37^ and the intramolecular iridium-catalyzed *O*-allylation of oximes.^38^

Despite the development of several synthetic strategies to synthesize isoxazoline, methods for the practical synthesis of optically active isoxazolines with high diastereoselectivities and enantioselectivities remain limited. Herein, we report a new protocol for the metal-free asymmetric synthesis of functionalized isoxazolines from the commercially available and simple starting materials: 2-methylfuran and (*S*)-epichlorohydrin.

## Results and Discussion

*All reaction conditions were developed first with racemic compounds. After optimization of the reaction conditions, the reactions were repeated with enantiopure compounds.*

**Scheme 1:**
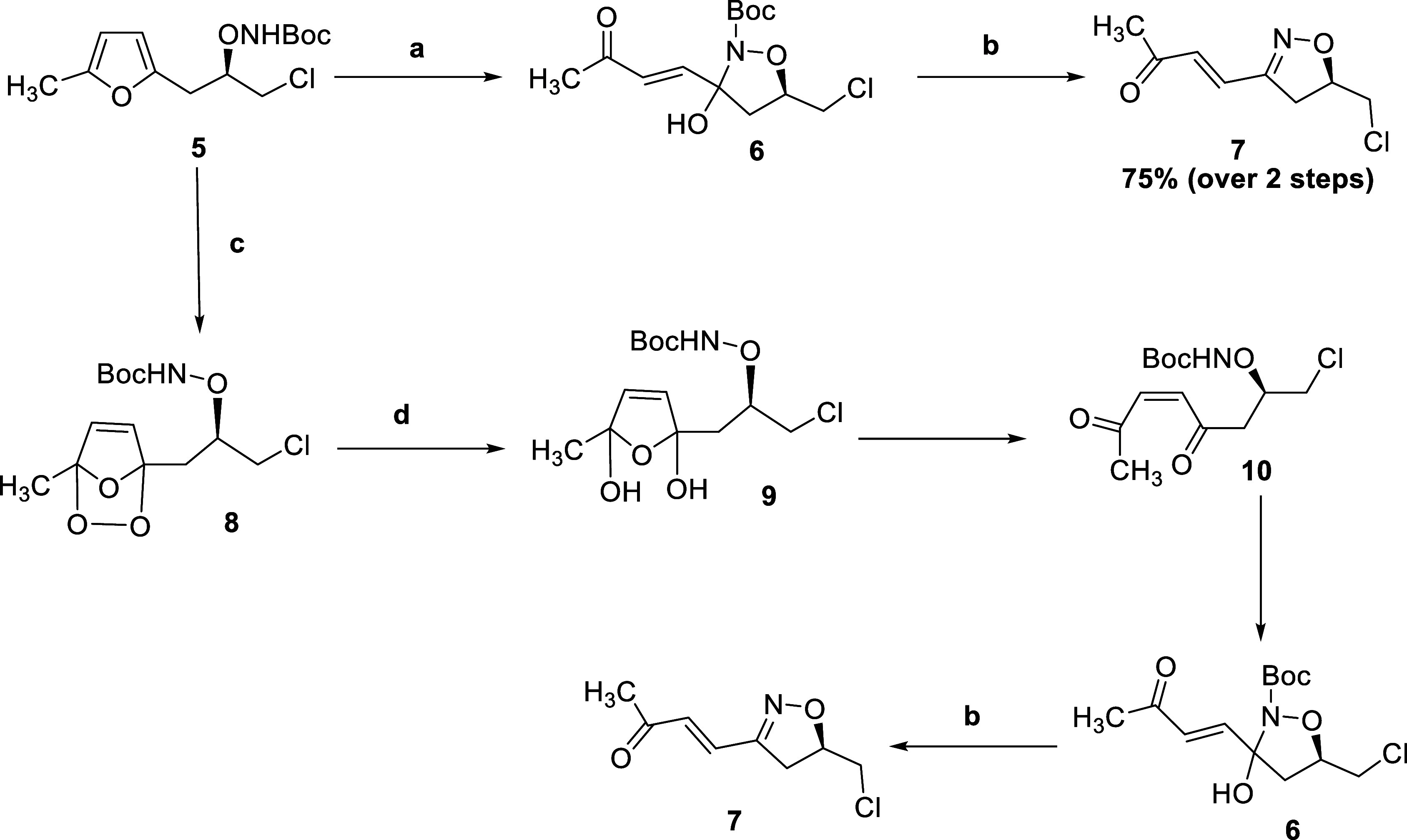
(a) *n*-BuLi, THF, −78 °C, Then (*S*)-Epichlorohydrin, BF_3_·OEt_2,_ −78 °C to rt, N_2_; (b) PPh_3_, NHPI, THF, DEAD, 0 °C to reflux, N_2_; (c) NH_2_NH_2_·*x*H_2_O, CH_2_Cl_2_, rt, N_2_; and (d) Et_3_N, Boc_2_O, THF, 0 °C, N_2_

Utilizing our previously reported reaction conditions, lithiation of 2-methyl furan was performed in THF using *n*-BuLi at −78 °C, followed by epoxide ring opening of *rac*-epichlorohydrin en route to **2** (Scheme 1).^39,40^ BF_3_·OEt_2_ was essential for promoting the epoxide ring opening but induced gelation of the reaction medium, complicating the isolation of **2** in high yield. The gelation was mitigated by carefully tuning the ratio of THF to BF_3_·OEt_2_ (120 mL/30.5 mmol).

Upon the successful synthesis of **2** (65% yield), we embarked on the synthesis of **3** via a Mitsunobu inversion.^39^ The mixture of **2**, triphenylphosphine, and *N*-hydroxyphthalimide (NHPI) in THF at 0 °C was treated with DEAD, allowed to warm to room temperature, and then heated at 60 °C for 16 h. After purification, **3** was obtained in 75% yield.

Treatment of **3** with the NH_2_NH_2_·*x*H_2_O in CH_2_Cl_2_ gave the hydroxylaminoalkyl furan **4** that was immediately protected with Boc_2_O and Et_3_N to afford **5** in 66% yield because of the instability of the hydroxylamino group. Unfortunately, to improve the yield of protection by tuning the amount of Et_3_N, adding DMAP as a cobase, and running the reaction at both 0 and 50 °C were unsuccessful.

The oxidation of furan was found to be significantly dependent on the substrate, prompting the evaluation of various chemical^41−44^ and photochemical oxidation conditions.^39,45,46^ To screen the oxidation conditions, a CH_2_Cl_2_ solution of **5** was treated with 1 equiv of *m*-CPBA (0 °C to room temperature, overnight). However, this did not lead to complete consumption of the furan. Increasing the equivalents of *m*-CPBA failed to cleanly oxidize the furan, so we sought recourse to photochemical oxidation. For this, a CH_2_Cl_2_ solution of **5** containing a catalytic amount of sensitizer (TPP) was irradiated with a sunlight lamp (250–500 W) while purging the solution with oxygen, followed by a reductive workup by adding Me_2_S. Unfortunately, none of the intermediates **10** and **6** or the target **7** were observed.

**Scheme 2:**
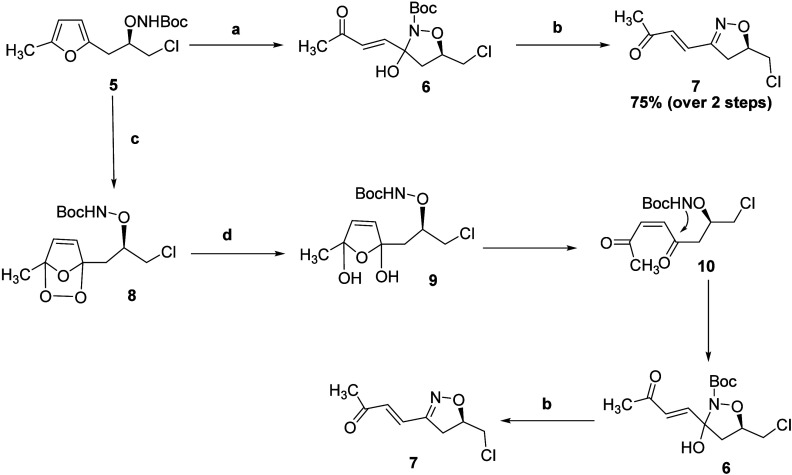
(a) NaHCO_3_, NaOAc, NBS, THF, 0 °C to rt; (b) *p-*TSA, CH_2_Cl_2_, rt; (c) O_2_, TPP, *hv*, CH_2_Cl_2_, 0 °C; and (d) Me_2_S

In contrast to the previous attempts, an NBS-mediated oxidation of **5** proved to be more successful. Treatment of **5** with 2 equiv of NBS and workup with a catalytic amount of *p*-TSA smoothly afforded the isoxazoline **7** in 54% yield. By adjusting the amount of NBS to 1.5 equiv and decreasing the time to 50 min, the yield of **7** was increased to 75% yield (Scheme 2). Scaling up the reaction to 3–4 mmol successfully afforded **7**, demonstrating excellent reproducibility across scales ranging from 60 mg to 2 g. The structural assignment of **7** was corroborated by ^1^H-, ^13^C NMR, and HRMS analysis.

**Scheme 3:**
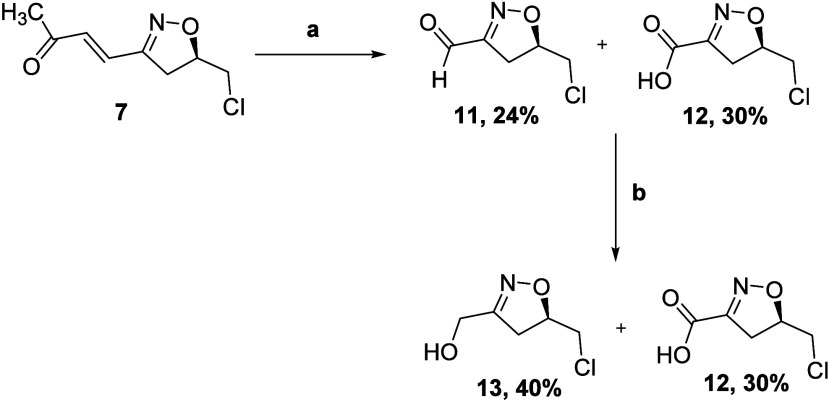
(a) RuCl_3_·*x*H_2_O, NaIO_4_, EtOAc, ACN, H_2_O, rt and (b) NaBH_4_, MeOH, 0 °C, and H^+^/H_2_O

Our next goal was to cleave the unsaturated double bond of **7** to access an isoxazoline that is appropriately functionalized for further manipulation. Employing a catalytic amount of RuCl_3_·*x*H_2_O (0.02 equiv) with NaIO_4_ as the stoichiometric oxidant (4.2 equiv) in a mixture of ethyl acetate, acetonitrile, and H_2_O afforded the isoxazolines **11** (24%) and **12** (30%) very cleanly but in a low yield (Scheme 3).

**Scheme 4:**
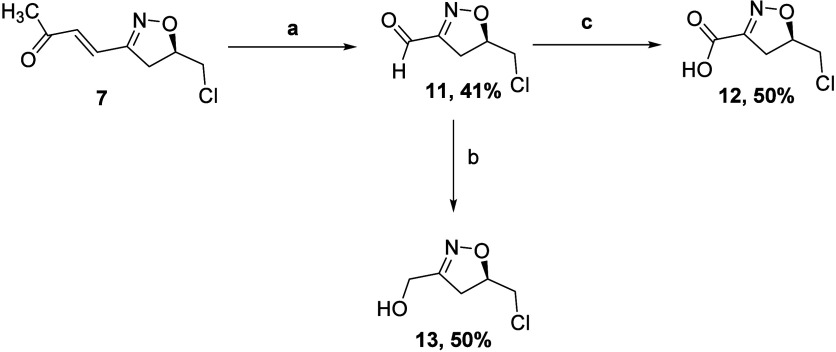
(a) RuCl_3_·*x*H_2_O, NaIO_4_, DCE, H_2_O, rt; (b) NaBH_4_, MeOH, 0 °C, and H^+^/H_2_O; and (c) Jones Reagent, Acetone, rt

We traced the problem to the volatility of aldehyde **11** and therefore repeated the oxidation and then added NaBH_4_ to reduce aldehyde **11***in situ* to the less-volatile alcohol **13**; purification on silica afforded **12** in 30% yield and **13** in 40% yield. Attempts to synthesize aldehyde **11** by oxidation with RuCl_3_·*x*H_2_O and Oxone were unsuccessful as the oxidation was very slow and the formation of **11** was negligible. Employing RuCl_3_·*x*H_2_O and NaIO_4_ in a biphasic mixture of DCE and H_2_O for 2 h afforded aldehyde **11** as the sole product in 41% yield (Scheme 4); again, a yield compromised by volatility. Oxidation followed by reduction with NaBH_4_ to convert **11** into alcohol **13** led to a 50% yield of the pure alcohol **13**. Alternatively, oxidation of the aldehyde **11***in situ* to the carboxylic acid under Jones oxidation conditions gave pure **12** in 50% yield over two consecutive steps (Scheme 4).

**Scheme 5:**
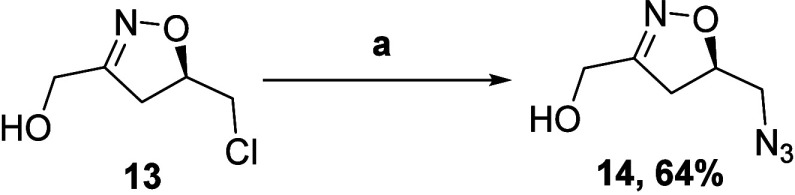
(a) NaN_3_, DMSO, 60 °C

Having succeeded in cleaving the unsaturated double bond, we sought to feature **13** as a key intermediate for nucleophilic displacement at the −CH_2_Cl site. Treating alcohol **13** with NaN_3_ in DMSO and heating to 60 °C for 36 h smoothly afforded **14** in 64% yield (Scheme 5).

**Scheme 6:**
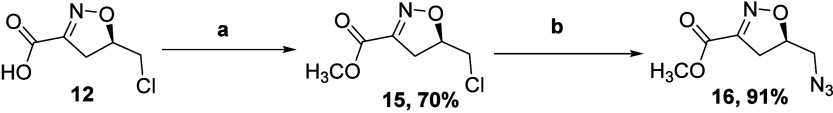
(a) SOCl_2_, MeOH, 0 °C to rt and (b) NaN_3_, DMSO, 60 °C

Converting the acid **12** to methyl ester **15** with MeOH and SOCl_2_ and treating the ester **15** with NaN_3_ in DMSO at 60 °C afforded, after purification, **16** in 91% yield (Scheme 6).

## Conclusion

In conclusion, a new and straightforward method was developed for the asymmetric synthesis of several 3,5-disubstituted isoxazolines that are succinctly functionalized for further manipulations. The hydroxylaminoalkyl furan was prepared from 2-methylfuran via a ring opening of (*S*)-epichlorohydrin, installation of the hydroxylamino group, and oxidation to the α,β-unsaturated dione with NBS. The oxidized intermediate was converted to the isoxazoline in the presence of a catalytic amount of *p-*TSA and transformed to isoxazolines bearing carboxylic acid, aldehyde, and alcohol functional groups. Having two sites for chemoselective functionalization on the isoxazoline allows for further derivatization, leading to potentially synthetically or biologically important target compounds.

We believe that this methodology will find useful applications allowing rapid and easy access to enantiopure isoxazoline motifs. This work represents one of the few examples in which furan-based hydroxylamine derivatives have been used for the synthesis of structurally complex heterocycles. The highly scalable nature of this novel approach enables the efficient incorporation of functionalized isoxazolines, making the sequences particularly valuable for large-scale synthesis campaigns and diverse medicinal chemistry applications. Further studies on the oxidation of hydroxylaminoalkyl furan to provide novel heterocycles will be reported in due course.

## Experimental Section

### General Information

All reactions were carried out using oven-dried glassware. All reagents and solvents were purchased from Sigma-Aldrich, Alfa Aesar, Merck depending on their availability and used without further purification. All reactions were monitored by TLC (Merck 105715 Silica Gel 60 F254 25 TLC Plates) with detection by UV light (254 nm). Vanillin, CAM and PMA were used as the TLC stains. The crude reaction mixtures were purified by column chromatography using Silica gel 60 (0.040–0.063 mm, 230–400 mesh). FT-IR spectra were recorded on a PerkinElmer Spectrum 100 spectrometer. Melting points were recorded on a Buchi B-545 melting point apparatus and are uncorrected. ^1^H and ^13^C NMR spectra were recorded on a Varian 500 MHz spectrometer in CDCl_3_ or DMSO-*d*_6_. Chemical shifts (δ) are quoted in ppm and referenced to TMS as internal standard. Coupling constants (*J*) are quoted in Hz. Multiplicity was recorded for ^1^H NMR as follows: *s* = singlet, *brs* = broad singlet, *d* = doublet, *t* = triplet, *q* = quartet, *m* = multiplet, *p* = pentet, *dd* = doublet of doublets. HRMS analyses were performed on Agilent Technologies 6200 series TOF/6500 series.

#### Synthesis of (*S*)*-*1-Chloro-3-(5-methylfuran-2-yl)propan-2-ol (**2**)

To a solution of 2-methyl furan (5 g, 60.9 mmol) in dry THF (100 mL) was added *n*-BuLi (2.5 M in hexanes, 60.9 mmol) at −78 °C under N_2_ atm. The resulting mixture was stirred for 2 h while warming to −30 °C. After 1 h at −30 °C, the reaction was cooled back to −78 °C and treated with a solution of (*S*)-epichlorohydrin (4.8 mL, 60.9 mmol) in dry THF (20 mL). Then, BF_3_·OEt_2_ (3.8 mL, 30.5 mmol) was added to the reaction mixture. The resulting mixture was allowed to warm to rt. After 2 h, the reaction was quenched with a saturated aqueous NH_4_Cl (30 mL) solution. The crude reaction mixture was extracted with EtOAc (3 × 35 mL), dried over Na_2_SO_4_, filtered, and concentrated *in vacuo.* The crude material was purified by column chromatography to afford **2** (6.90 g, 65%) as a light yellow oil. **R**_***f***_: 0.45 (20%, EtOAc/Hex); **[α]**_**D**_^**25**^: 8.77 (c = 0.57, EtOAc); **FTIR** (ATR): ν = 3392 cm^–1^; ^**1**^**H NMR** (500 MHz, CDCl_3_): δ 6.01 (d, *J* = 2.8 Hz, 1H), 5.87 (d, *J* = 1.9 Hz, 1H), 4.09 (m, 1H), 3.62 (dd, *J* = 11.2, 4.2 Hz, 1H), 3.52 (dd, *J* = 11.2, 6.2 Hz, 1H), 2.87 (d, *J* = 6.4 Hz, 2H), 2.52 (s, 1H), 2.25 (s, 3H) ppm; ^**13**^**C NMR** (126 MHz, CDCl_3_): δ 151.6, 149.3, 108.4, 106.3, 70.3, 49.0, 33.2, 13.6 ppm; **HRMS** (ESI): *m*/*z* calcd for C_8_H_11_ClO_2_ [M+H^+^]: 177.0496, found 177.0484.

#### Synthesis of (*R*)-2-((1-Chloro-3-(5-methylfuran-2-yl)propan-2-yl)oxy)isoindoline-1,3-dione (**3**)

To a solution of triphenylphospine (9.2 g, 35.1 mmol) and *N*-hydroxyphthalimide (5.7 g, 35.1 mmol) in dry THF (80 mL) was added a solution of **2** (2.05 g, 11.7 mmol) in dry THF (20 mL) under N_2_ atm at 0 °C. Then, DEAD (40 wt % in toluene, 6.4 mL, 35.1 mmol) was added dropwise to the reaction mixture. The reaction was stirred at rt for 2 h and then refluxed for 8 h. Upon completion, judged by TLC, the reaction was diluted with saturated brine (20 mL) and extracted with EtOAc (3 × 30 mL), dried over Na_2_SO_4_, filtered, and concentrated *in vacuo.* The crude product was purified by column chromatography to afford **3** (2.80 g, 75%) as a white solid. **R**_***f***_: 0.44 (20%, EtOAc/Hex); **m.p.** 109–110 °C; **[α]**_**D**_^**25**^: 16.5 (c = 0.61, EtOAc); **FTIR** (ATR): ν = 3103, 2943, 1789, 1732, 1715 cm^–1^; ^**1**^**H NMR** (500 MHz, CDCl_3_): δ 7.86–7.82 (m, 2H), 7.77–7.75 (m, 2H), 6.11 (m, 1H), 5.84 (m, 1H), 4.71 (m, 1H), 3.85 (dd, *J* = 12.1, 4.8 Hz, 1H), 3.75 (dd, *J* = 12.1, 4.4 Hz, 1H), 3.26–3.18 (m, 2H), 2.20 (s, 3H) ppm; ^**13**^**C NMR** (126 MHz, CDCl_3_): δ 163.9, 151.6, 147.9, 134.8, 129.0, 123.8, 108.9, 106.4, 85.2, 44.1, 30.0, 13.6 ppm; **HRMS** (ESI): *m*/*z* calcd for C_16_H_14_ClNO_4_ [M+H^+^]: 320.0690, found 320.0669.

#### Synthesis of (*R*)-*O*-(1-Chloro-3-(5-methylfuran-2-yl)propan-2-yl)hydroxylamine (**4**)

To a solution of **3** (4.8 g, 15 mmol) in dry CH_2_Cl_2_ (80 mL) was added hydrazine hydrate (3.6 mL, 75 mmol) under N_2_ atm at rt. The reaction mixture was stirred for 12 h. Upon completion, the reaction was judged by TLC, and the mixture was filtered and concentrated *in vacuo*. The crude product **4** (2.77 g, 94% as yellow oil) was used as is in the next step without purification. ^**1**^**H NMR** (500 MHz, CDCl_3_): δ 5.98 (s, 1H), 5.87 (s, 1H), 5.41 (s, 2H), 4.03 (m, 1H), 3.77 (m, 1H), 3.63 (m, 1H), 2.97–2.87 (m, 2H), 2.26 (s, 3H) ppm; ^**13**^**C NMR** (126 MHz, CDCl_3_): δ 151.2, 149.6, 107.8, 106.2, 81.3, 44.6, 29.5, 13.6 ppm.

#### Synthesis of *tert*-Butyl-(*R*)-((1-chloro-3-(5-methylfuran-2-yl)propan-2-yl)oxy)carbamate (**5**)

To a solution of crude **4** (2.23 g, 11.4 mmol) in dry THF (100 mL) was added Et_3_N (3.96 mL, 28.5 mmol) under N_2_ atm at 0 °C. Boc_2_O (3.73 g, 17.1 mmol) in dry THF (20 mL) was then added and the reaction stirred overnight. The reaction was quenched with saturated aqueous NH_4_Cl (20 mL) and extracted with EtOAc (3 × 30 mL), dried over Na_2_SO_4_, filtered, and concentrated *in vacuo.* The crude product was purified via radial chromatography to afford **5** (2.50 g, 66%) as a colorless oil. **R**_***f***_: 0.5 (20%, EtOAc/Hex); **[α]**_**D**_^**25**^: 30.9 (c = 0.57, EtOAc); **FTIR** (ATR): ν = 3296, 1701 cm^–1^; ^**1**^**H NMR** (500 MHz, CDCl_3_): δ 5.91 (m, 1H), 5.74 (m, 1H), 4.12 (m, 1H), 3.62 (dd, *J* = 11.8, 4.8 Hz, 1H), 3.50 (dd, *J* = 11.8, 4.3 Hz, 1H), 2.92 (dd, *J* = 15.3, 5.9 Hz, 1H), 2.86 (dd, *J* = 15.3, 7.2 Hz, 1H), 2.12 (s, 3H), 1.36 (s, 9H) ppm; ^**13**^**C NMR** (126 MHz, CDCl_3_): δ 157.2, 151.2, 148.8, 108.3, 106.2, 82.7, 82.1, 44.0, 29.2, 28.2, 13.5 ppm; **HRMS** (ESI): *m*/*z* calcd for C_13_H_20_ClNO_4_ [M+H^+^]: 290.1159, found 290.1143.

#### Synthesis of (*R*,*E*)-4-(5-(Chloromethyl)-4,5-dihydroisoxazol-3-yl)but-3-en-2-one (**7**)

To a solution of **5** (1.32 g, 4.5 mmol) in a 2:1 mixture of THF/H_2_O (140 mL) at 0 °C was added NaHCO_3_ (267 mg, 11.2 mmol), NaOAc (538 mg, 6.7 mmol), and NBS (1.19 g, 6.7 mmol) sequentially. The resulting mixture was allowed to warm to rt and stirred for 2 h. Upon completion, judged by TLC, the reaction was quenched with a saturated NaHCO_3_ solution (20 mL) and concentrated *in vacuo*. The residue was then diluted with brine (10 mL) and extracted with EtOAc (3 × 20 mL), dried over Na_2_SO_4_, filtered, and concentrated *in vacuo*. The resulting crude **6** was used directly in the next step without purification. Crude **6** was dissolved in CH_2_Cl_2_ (50 mL) and treated with *p-*TSA (137 mg). The resulting mixture was stirred overnight and quenched with saturated NH_4_Cl (10 mL). Then, the mixture was extracted with CH_2_Cl_2_ (3 × 10 mL), the organic layer was separated, dried over Na_2_SO_4_, filtered, and concentrated *in vacuo.* The crude oil was purified by column chromatography to afford **7** (626 mg, 75% over 2 steps) as a white solid. **R**_***f***_: 0.21 (30%, EtOAc/Hex); **m.p.** 89–91 °C; **[α]**_**D**_^**25**^: −190 (c = 0.5, EtOAc); **FTIR** (ATR): ν = 1697 cm^–1^; ^**1**^**H NMR** (500 MHz, CDCl_3_): δ 7.37 (d, *J* = 16.5 Hz, 1H), 6.28 (d, *J* = 16.5 Hz, 1H), 5.02 (m, 1H), 3.69 (dd, *J* = 11.6, 4.1 Hz, 1H), 3.59 (dd, *J* = 11.6, 6.7 Hz, 1H), 3.24 (dd, *J* = 16.9, 10.8 Hz, 1H), 3.10 (dd, *J* = 16.9, 6.8 Hz, 1H), 2.36 (s, 3H) ppm; ^**13**^**C NMR** (126 MHz, CDCl_3_): δ 197.7, 156.3, 134.4, 131.4, 81.2, 44.8, 36.3, 27.2 ppm; **HRMS** (ESI): *m*/*z* calcd for C_8_H_10_ClNO_2_ [M+H^+^]: 188.0478, found 188.0475.

#### Synthesis of (*R*)-5-(Chloromethyl)-4,5-dihydroisoxazole-3-carbaldehyde (**11**), (*R*)-5-(Chloromethyl)-4,5-dihydroisoxazole-3-carboxylate (**12**)

RuCl_3_·*x*H_2_O (4.9 mg, 0.02 mmol), NaIO_4_ (1.08 g, 5.0 mmol) and isoxazoline **7** (220 mg, 1.2 mmol) were suspended in a 1:1:2 mixture of EtOAc/ACN/H_2_O (20 mL) at rt. The reaction mixture was stirred at room temperature for 2 h. The pH was adjusted to 2 with 1 M HCl and extracted with EtOAc (3 × 5 mL). The combined organic layers were dried over Na_2_SO_4_, filtered, and concentrated *in vacuo.* The crude oil was purified by column chromatography to afford **11** (42 mg, 24%) as a light yellow oil and **12** (59 mg, 30%) as a white solid. **(*R*)-5-(Chloromethyl)-4,5-dihydroisoxazole-3-carbaldehyde (11)**: **R**_***f***_: 0.75 (50%, EtOAc/Hex); ^**1**^**H NMR** (500 MHz, CDCl_3_): δ 9.90 (s, 1H), 5.10 (m, 1H), 3.70–3. 63 (m, 2H), 3.23 (dd, *J* = 17.8, 11.2 Hz, 1H), 3.12 (dd, *J* = 17.8, 7.3 Hz, 1H) ppm; ^**13**^**C NMR** (126 MHz, CDCl_3_): δ 185.3, 159.0, 82.9, 44.8, 33.9 ppm. **(*****R*****)*****-*****5-(Chloromethyl)-4,5-dihydroisoxazole-3-carboxylate (12)**: **R**_***f***_: 0.13 (70%, EtOAc/Hex); **m.p.** 96–98 °C; **[α]**_**D**_^**25**^: −156.25 (c = 0.32, EtOAc); **FTIR** (ATR): ν = 2985, 1717 cm^–1^; ^**1**^**H NMR** (500 MHz, CDCl_3_): δ 6.57 (s, 1H), 5.14 (m, 1H), 3.72–3.64 (m, 2H), 3.37 (dd, *J* = 18.0, 11.2 Hz, 1H), 3.24 (dd, *J* = 18.0, 7.3 Hz, 1H) ppm; ^**13**^**C NMR** (126 MHz, CDCl_3_): δ 163.1, 151.1, 83.0, 44.6, 36.4 ppm.

#### Synthesis of (*R*)-5-(Chloromethyl)-4,5-dihydroisoxazole-3-carboxylate (**12**) and (*R*)-(5-(Chloromethyl)-4,5-dihydroisoxazol-3-yl)methanol (**13**)

RuCl_3_·*x*H_2_O (10.4 mg, 0.05 mmol), NaIO_4_ (2.4 g, 11.3 mmol) and isoxazoline **7** (500 mg, 2.7 mmol) were suspended in a 1:1:2 mixture of EtOAc/ACN/H_2_O (40 mL) at rt. The reaction mixture was stirred at room temperature for 2 h. The pH was adjusted to 2 with 1 M HCl and extracted with EtOAc (3 × 5 mL). The combined organic layers were dried over Na_2_SO_4_, filtered, and concentrated *in vacuo.* The crude mixture was used in the next step without further purification. The crude was dissolved in MeOH (20 mL) and treated with NaBH_4_ (204 mg, 5.4 mmol) at 0 °C. The resulting mixture was stirred for 30 min. The reaction was quenched with a saturated NH_4_Cl (5 mL) solution and extracted with EtOAc (3 × 5 mL). The combined organic layers were dried over Na_2_SO_4_, filtered, and concentrated *in vacuo.* The crude oil was purified by column chromatography to afford **13** (160 mg, 40%) as a light yellow oil. The aqueous layer was acidified to pH 2 with 1 M HCl then extracted with EtOAc (3 × 5 mL), dried over Na_2_SO_4_, filtered, and concentrated *in vacuo.* The residue was purified by crystallization to give **12** (131 mg, 30%) as a white solid. **(*****R*****)*****-*****5-(Chloromethyl)-4,5-dihydroisoxazole-3-carboxylate (12)**: **R**_***f***_: 0.13 (70%, EtOAc/Hex); **m.p.** 96–98 °C; **[α]**_**D**_^**25**^: −156.25 (c = 0.32, EtOAc); **FTIR** (ATR): ν = 2985, 1717 cm^–1^; ^**1**^**H NMR** (500 MHz, CDCl_3_): δ 6.57 (s, 1H), 5.14 (m, 1H), 3.72–3.64 (m, 2H), 3.37 (dd, *J* = 18.0, 11.2 Hz, 1H), 3.24 (dd, *J* = 18.0, 7.3 Hz, 1H) ppm; ^**13**^**C NMR** (126 MHz, CDCl_3_): δ 163.1, 151.1, 83.0, 44.6, 36.4 ppm. **(*****R*****)-(5-(Chloromethyl)-4,5-dihydroisoxazol-3-yl)methanol (13)**: **R**_***f***_**:** 0.36 (50%, EtOAc/Hex); **[α]**_**D**_^**25**^: −34.8 (c = 1.7, EtOAc); **FTIR** (ATR): ν = 3368 cm^–1^; ^**1**^**H NMR** (500 MHz, CDCl_3_): δ 4.87 (m, 1H), 4.39 (s, 2H), 3.62 (dd, *J* = 11.3, 4.3 Hz, 1H), 3.54 (dd, *J* = 11.3, 6.6 Hz, 1H), 3.21 (dd, *J* = 17.4, 10.7 Hz, 1H), 3.03 (dd, *J* = 17.4, 6.2 Hz, 1H), 2.87 (s, 1H) ppm; ^**13**^**C NMR** (126 MHz, CDCl_3_): δ 158.6, 79.5, 57.9, 45.1, 38.6 ppm; **HRMS** (ESI): *m*/*z* calcd for C_5_H_8_ClNO_2_ [M+H^+^]: 150.0322, found 150.0313.

#### Synthesis of (*R*)*-*5-(Chloromethyl)-4,5-dihydroisoxazole-3-carbaldehyde (**11**)

RuCl_3_·*x*H_2_O (16.5 mg, 0.08 mmol) and **7** (205 mg, 1.1 mmol) were suspended in a 3:2 mixture of DCE/H_2_O (10 mL) at rt. NaIO_4_ (941.1 mg, 4.4 mmol) in a 3:2 mixture of DCE/H_2_O (10 mL) was added to the reaction within 5 min. The reaction was stirred for 2 h and upon completion, judged by TLC, the reaction was quenched with saturated aqueous Na_2_S_2_O_3_ (5 mL) solution, and the layers were separated. The aqueous layer was extracted with EtOAc (3 × 5 mL). The crude oil was purified by column chromatography to afford **11** (66 mg, 41%) as a light yellow oil. **(*****R*****)-5-(Chloromethyl)-4,5-dihydroisoxazole-3-carbaldehyde (11)**: **R**_***f***_: 0.75 (50%, EtOAc/Hex); ^**1**^**H NMR** (500 MHz, CDCl_3_): δ 9.90 (s, 1H), 5.10 (m, 1H), 3.70–3.63 (m, 2H), 3.23 (dd, *J* = 17.8, 11.2 Hz, 1H), 3.12 (dd, *J* = 17.8, 7.3 Hz, 1H) ppm; ^**13**^**C NMR** (126 MHz, CDCl_3_): δ 185.3, 159.0, 82.9, 44.8, 33.9 ppm.

#### Synthesis of (*R*)-5-(Chloromethyl)-4,5-dihydroisoxazole-3-carbaldehyde (**13**)

RuCl_3_·*x*H_2_O (16.6 mg, 0.08 mmol) and **7** (214 mg, 1.14 mmol) were suspended in a 3:2 mixture of DCE/H_2_O (10 mL) at rt. NaIO_4_ (975.8 mg, 4.6 mmol) in a 3:2 mixture of DCE/H_2_O (10 mL) was added to the reaction within 5 min. The reaction was stirred for 2 h and upon completion, judged by TLC, the reaction was quenched with a saturated aqueous Na_2_S_2_O_3_ (10 mL) solution, and the layers were separated. The aqueous layer was extracted with EtOAc (3 × 15 mL). The crude mixture was used in the next step without further purification. The crude was dissolved in MeOH (20 mL) and then treated with NaBH_4_ (87.0 mg, 2.3 mmol) at 0 °C. The resulting mixture was stirred for 30 min. The reaction was quenched with a saturated aqueous NH_4_Cl (5 mL) solution and extracted with EtOAc (3 × 10 mL). The combined organic layers were dried over Na_2_SO_4_, filtered, and concentrated *in vacuo.* The crude oil was purified by column chromatography to afford **13** (85 mg, 50%) as a light yellow oil. **R**_***f***_: 0.36 (50%, EtOAc/Hex); **[α]**_**D**_^**25**^: −34.8 (c = 1.7, EtOAc); **FTIR** (ATR): ν = 3368 cm^–1^; ^**1**^**H NMR** (500 MHz, CDCl_3_): δ 4.87 (m, 1H), 4.39 (s, 2H), 3.62 (dd, *J* = 11.3, 4.3 Hz, 1H), 3.54 (dd, *J* = 11.3, 6.6 Hz, 1H), 3.21 (dd, *J* = 17.4, 10.7 Hz, 1H), 3.03 (dd, *J* = 17.4, 6.2 Hz, 1H), 2.87 (s, 1H) ppm; ^**13**^**C NMR** (126 MHz, CDCl_3_): δ 158.6, 79.5, 57.9, 45.1, 38.6 ppm; **HRMS** (ESI): *m*/*z* calcd for C_5_H_8_ClNO_2_ [M+H^+^]: 150.0322, found 150.0313.

#### Synthesis of (*R*)*-*5-(Chloromethyl)-4,5-dihydroisoxazole-3-carboxylate (**12**)

RuCl_3_·*x*H_2_O (14.5 mg, 0.07 mmol) and **7** (195 mg, 1.04 mmol) were suspended in a 3:2 mixture of DCE/H_2_O (14 mL) at rt. NaIO_4_ (889.3 mg, 4.2 mmol) in a 3:2 mixture of DCE/H_2_O (14 mL) was added to the reaction within 5 min. The reaction was stirred for 2 h and upon completion, judged by TLC, the reaction was quenched with a saturated aqueous Na_2_S_2_O_3_ (14 mL) solution, and the layers were separated. The aqueous layer was extracted with EtOAc (3 × 15 mL). The crude mixture was used in the next step without further purification. To a solution of **11** (153.5 mg, 1.04 mmol) in acetone (20 mL) was added Jones reagent (1.34 M, 1.55 mL) at rt. Upon completion, judged by TLC in 30 min, the reaction mixture was concentrated *in vacuo*. The residue was mixed with brine (5 mL) and EtOAc (10 mL), and once separated, the aqueous layer was extracted with EtOAc (2 × 10 mL). The combined organic layers were dried over Na_2_SO_4_, filtered, and concentrated *in vacuo.* The crude material was purified by precipitation using EtOAc/Hex to afford **12** (85 mg, 50%) as a white solid. **R**_***f***_: 0.13 (70%, EtOAc/Hex); **m.p.** 96–98 °C; **[α]**_**D**_^**25**^: −156.25 (c = 0.32, EtOAc); **FTIR** (ATR): ν = 2985, 1717 cm^–1^; ^**1**^**H NMR** (500 MHz, CDCl_3_): δ 6.57 (s, 1H), 5.14 (m, 1H), 3.72–3.64 (m, 2H), 3.37 (dd, *J* = 18.0, 11.2 Hz, 1H), 3.24 (dd, *J* = 18.0, 7.3 Hz, 1H) ppm; ^**13**^**C NMR** (126 MHz, CDCl_3_): δ 163.1, 151.1, 83.0, 44.6, 36.4 ppm.

#### Synthesis of (*R*)*-*(5-(Azidomethyl)-4,5-dihydroisoxazol-3-yl)methanol (**14**)

A mixture of NaN_3_ (507.1 mg, 7.8 mmol) and **13** (194.4 mg, 1.3 mmol) in DMSO (2 mL) was heated at 60 °C for 36 h under N_2_ atm. Upon completion, judged by TLC, the reaction was quenched with cold water (10 mL). The reaction was then extracted with EtOAc (3 × 20 mL) and washed with brine. The organic layer was dried over Na_2_SO_4_, filtered and concentrated *in vacuo*. The crude product was purified by column chromatography (50%, EtOAc/Hex) to afford **14** (130 mg, 64%) as a light brown oil. **R**_***f***_: 0.13 (50%, EtOAc/Hex); **[α]**_**D**_^**25**^: −211.5 (c = 0.4, EtOAc); **FTIR** (ATR): ν = 3384, 2103 cm^–1^; ^**1**^**H NMR** (500 MHz, CDCl_3_): δ 4.80 (m, 1H), 4.41 (s, 2H), 3.48 (dd, *J* = 13.1, 4.1 Hz, 1H), 3.35 (dd, *J* = 13.1, 5 Hz, 1H), 3.16 (dd, *J* = 17.4, 10.8 Hz, 1H), 2.91 (dd, *J* = 17.4, 7 Hz, 1H), 2.54 (brs, 1H) ppm; ^**13**^**C NMR** (126 MHz, CDCl_3_): δ 158.8, 79.0, 58.0, 53.5, 37.9 ppm; **HRMS** (ESI): *m*/*z* calcd for C_5_H_8_N_4_O_2_ [M+H^+^]: 157.0725, found 157.0717.

#### Synthesis of Methyl (*R*)-5-(Chloromethyl)-4,5-dihydroisoxazole-3-carboxylate (**15**)

To a solution of **12** (261.7 mg, 1.6 mmol) in dry MeOH (15 mL) was added dropwise SOCl_2_ (0.12 mL, 1.6 mmol) at 0 °C. The reaction mixture was allowed to warm to room temperature and stirred until the starting material was fully consumed as judged by TLC. The reaction mixture was neutralized with a saturated aqueous Na_2_CO_3_ solution and concentrated *in vacuo.* The residue was extracted with CH_2_Cl_2_ (3 × 10 mL) and the combined organic layers were washed with brine (10 mL), dried over Na_2_SO_4_, and concentrated *in vacuo*. The crude product was purified by column chromatography to afford **15** (199 mg, 70%) as a light yellow oil. **R**_***f***_: 0.59 (50%, EtOAc/Hex); **[α]**_**D**_^**25**^: −66.2 (c = 0.68, EtOAc); **FTIR** (ATR): ν = 1724 cm^–^1; ^**1**^**H NMR** (500 MHz, CDCl_3_): δ 5.06 (m, 1H), 3.89 (s, 3H), 3.67 (dd, *J* = 11.7, 4.1 Hz, 1H), 3.61 (dd, *J* = 11.7, 6.4 Hz, 1H), 3.35 (dd, *J* = 18.0, 11.2 Hz, 1H), 3.22 (dd, *J* = 18.0, 7.2 Hz, 1H) ppm; ^**13**^**C NMR** (126 MHz, CDCl_3_): δ 160.8, 151.2, 82.1, 53.1, 44.6, 37.1 ppm; **HRMS** (ESI): *m*/*z* calcd for C_6_H_8_NO_3_ [M+H^+^]: 178.0271, found 178.0265.

#### Synthesis of Methyl (*R*)*-*5-(Azidomethyl)-4,5-dihydroisoxazole-3-carboxylate (**16**)

A solution of **15** (178 mg, 1 mmol) in DMSO (4 mL) was treated with NaN_3_ (325.8 mg, 5.0 mmol). The reaction mixture was heated at 60 °C for 36 h under N_2_ atm. Upon completion, judged by TLC, the reaction was quenched with cold water (5 mL) then extracted with EtOAc (3 × 10 mL) and washed with brine. The combined organic layers were dried over Na_2_SO_4_, and concentrated *in vacuo*. The crude product was purified by column chromatography (50%, EtOAc/Hex) to afford **16** (168 mg, 91%) as a light brown oil. **R**_***f***_: 0.38 (50%, EtOAc/Hex); **[α]**_**D**_^**25**^: −100.8 (c = 0.62, EtOAc); **FTIR** (ATR): ν = 1724, 2102 cm^–1^; ^**1**^**H NMR** (500 MHz, CDCl_3_): δ 5.10 (m, 1H), 3.90 (s, 3H), 3.67 (qd, *J* = 11.7, 5.1 Hz, 2H), 3.37 (dd, *J* = 18.0, 11.2 Hz, 1H), 3.22 (dd, *J* = 18.0, 7.3 Hz, 1H) ppm; ^**13**^**C NMR** (126 MHz, CDCl_3_): δ 159.8, 150.2, 81.0, 76.4, 76.2, 76.0, 52.1, 43.6, 36.1 ppm; **HRMS** (ESI): *m*/*z* calcd for C_6_H_8_N_4_O_3_ [M+H^+^]: 185.0675, found 185.0668.

## Supporting Information Available

NMR spectra for all synthesized compounds (PDF)
