# Supplementary material for: Asymmetric Synthesis of Functionalized 2-Isoxazolines
Source: ACS Omega. 2025 Feb 12;10(7):6663–70. doi: 10.1021/acsomega.4c08062 (PMC11866192; doi:10.1021/acsomega.4c08062)
Supplement: Supplementary file 1 — ao4c08062_si_001.pdf [file ao4c08062_si_001.pdf]

Supporting Information

for

Asymmetric Synthesis of Functionalized 2-Isoxazolines

Beyza Hamur [a], Fatma Albayrak Halac[a], Fethiye Yilmazer [a], Fraser F. Fleming [b], Irem Kulu[a], and Ramazan Altundas[a]*

[a] Department of Chemistry, College of Science, Gebze Technical University, 41400 Gebze, Kocaeli, TR

[b] Department of Chemistry, Drexel University, 306 Disque Hall, Philadelphia PA 19104, USA

* Corresponding author. Email: raltundas@gtu.edu.tr

Table of Contents

1. **^{1}H NMR and ^{13}C NMR Spectra** 2

Figure S1. ^{1}H NMR spectrum of *(S)*-1-chloro-3-(5-methylfuran-2-yl)propan-2-ol (2)............4

Figure S2. ^{13}C NMR spectrum of *(S)*-1-chloro-3-(5-methylfuran-2-yl)propan-2-ol (2). 4

Figure S3. ^{1}H NMR spectrum of *(R)*-2-((1-chloro-3-(5-methylfuran-2-yl)propan-2-yl)oxy)isoindoline-1,3-dione (3)...5

Figure S4. ^{13}C NMR spectrum of *(R)*-2-((1-chloro-3-(5-methylfuran-2-yl)propan-2-yl)oxy)isoindoline-1,3-dione (3)...5

Figure S5. ^{1}H NMR spectrum of *(R)*-O-(1-chloro-3-(5-methylfuran-2-yl)propan-2-yl)hydroxylamine (4). 6

Figure S6. ^{13}C NMR spectrum of *(R)*-O-(1-chloro-3-(5-methylfuran-2-yl)propan-2-yl)hydroxylamine (4). 6

Figure S7. ^{1}H NMR spectrum of *tert*-butyl-*(R)*-((1-chloro-3-(5-methylfuran-2-yl)propan-2-yl)oxy)carbamate (5)...7

Figure S8. ^{13}C NMR spectrum of *tert*-butyl-*(R)*-((1-chloro-3-(5-methylfuran-2-yl)propan-2-yl)oxy)carbamate (5)...7

Figure S9. ^{1}H NMR spectrum of *(R,E)*-4-(5-(chloromethyl)-4,5-dihydroisoxazol-3-yl)but-3-en-2-one (7)...8

Figure S10. ^{13}C NMR spectrum of *(R,E)*-4-(5-(chloromethyl)-4,5-dihydroisoxazol-3-yl)but-3-en-2-one (7)...8

Figure S11. ^{1}H NMR spectrum of *(R)*-5-(chloromethyl)-4,5-dihydroisoxazole-3-carbaldehyde (11)...9

Figure S12. ^{13}C NMR spectrum of *(R)*-5-(chloromethyl)-4,5-dihydroisoxazole-3-carbaldehyde (11)...9

Figure S13. ^{1}H NMR spectrum of *(R)*-5-(chloromethyl)-4,5-dihydroisoxazole-3-carboxylate (12)...10

Figure S14. ^{13}C NMR spectrum of *(R)*-5-(chloromethyl)-4,5-dihydroisoxazole-3-carboxylate (12)...10

Figure S15. ^{1}H NMR spectrum of *(R)*-(5-(chloromethyl)-4,5-dihydroisoxazol-3-yl)methanol (13)...11

Figure S16. ^{13}C NMR spectrum of *(R)*-(5-(chloromethyl)-4,5-dihydroisoxazol-3-yl)methanol (13)...11

Figure S17. ^{1}H NMR spectrum of *(R)*-(5-(azidomethyl)-4,5-dihydroisoxazol-3-yl)methanol (14)...12

Figure S18. ^{13}C NMR spectrum of *(R)*-(5-(azidomethyl)-4,5-dihydroisoxazol-3-yl)methanol (14)...12

Figure S19. ^{1}H NMR spectrum of methyl *(R)*-5-(chloromethyl)-4,5-dihydroisoxazole-3-carboxylate (15). 13

Figure S20. ^{13}C NMR spectrum of methyl *(R)*-5-(chloromethyl)-4,5-dihydroisoxazole-3-carboxylate (15). 13

Figure S21. ^{1}H NMR spectrum of methyl *(R)*-5-(azidomethyl)-4,5-dihydroisoxazole-3-carboxylate (16). 14

Figure S22. ^{13}C NMR spectrum of methyl *(R)*-5-(azidomethyl)-4,5-dihydroisoxazole-3-carboxylate (16). 14

1. ^{1}H NMR and ^{13}C NMR Spectra

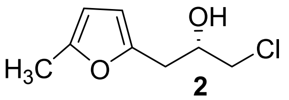

2

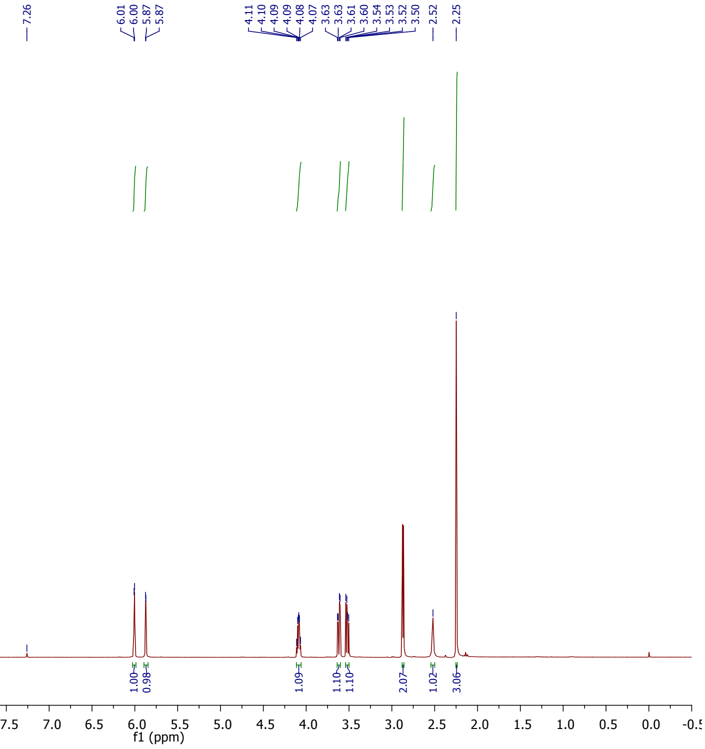

Figure S1. ^{1}H NMR spectrum of *(S)*-1-chloro-3-(5-methylfuran-2-yl)propan-2-ol (2).

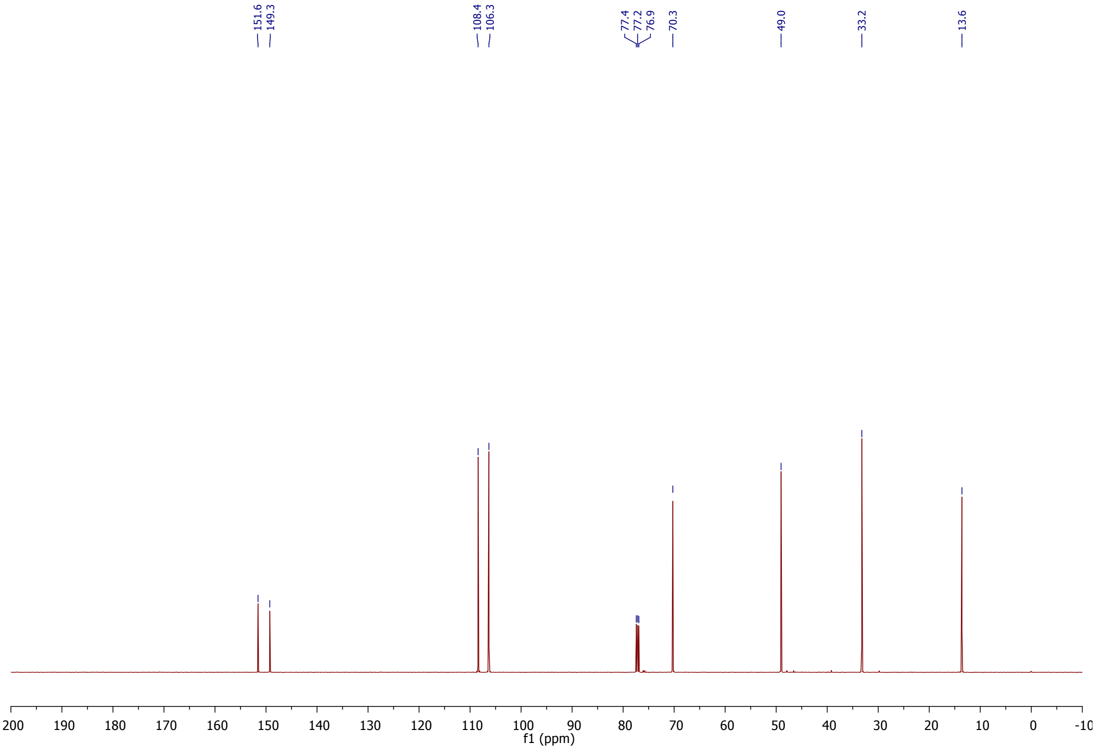

Figure S2. ^{13}C NMR spectrum of *(S)*-1-chloro-3-(5-methylfuran-2-yl)propan-2-ol (2).

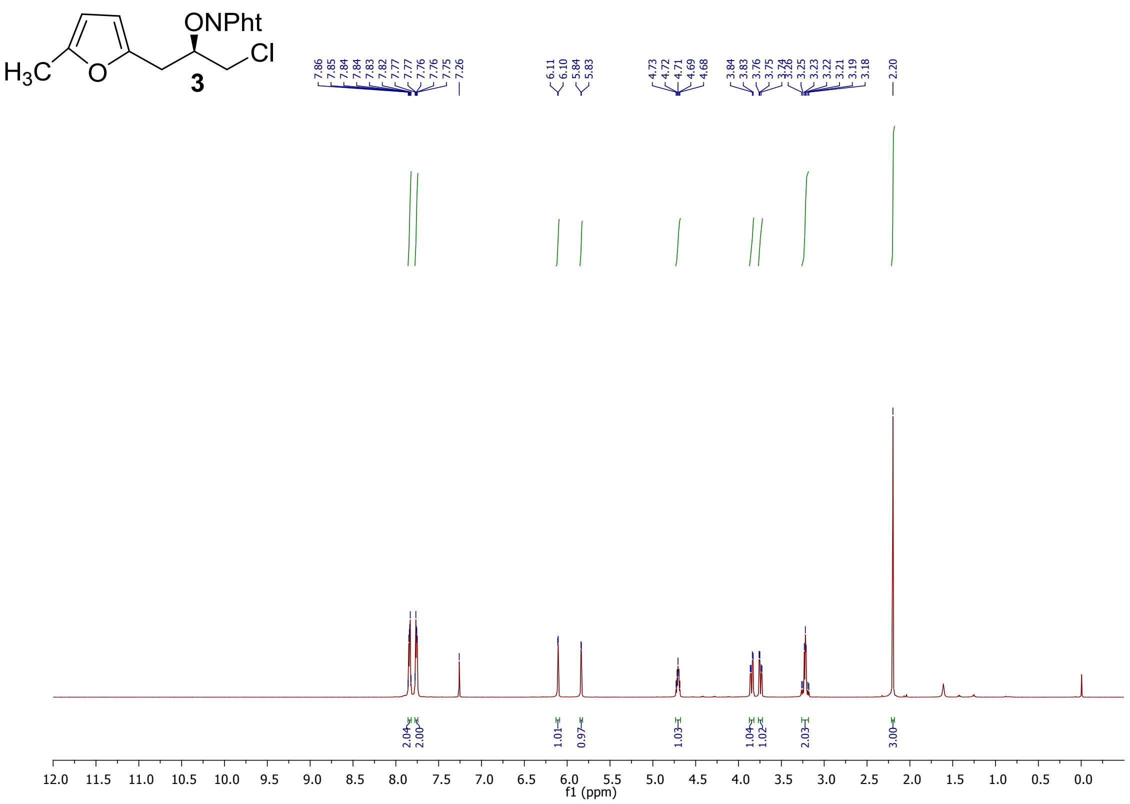

Figure S3. ^{1}H NMR spectrum of *(R)*-2-((1-chloro-3-(5-methylfuran-2-yl)propan-2-yl)oxy)isoindoline-1,3-dione (3).

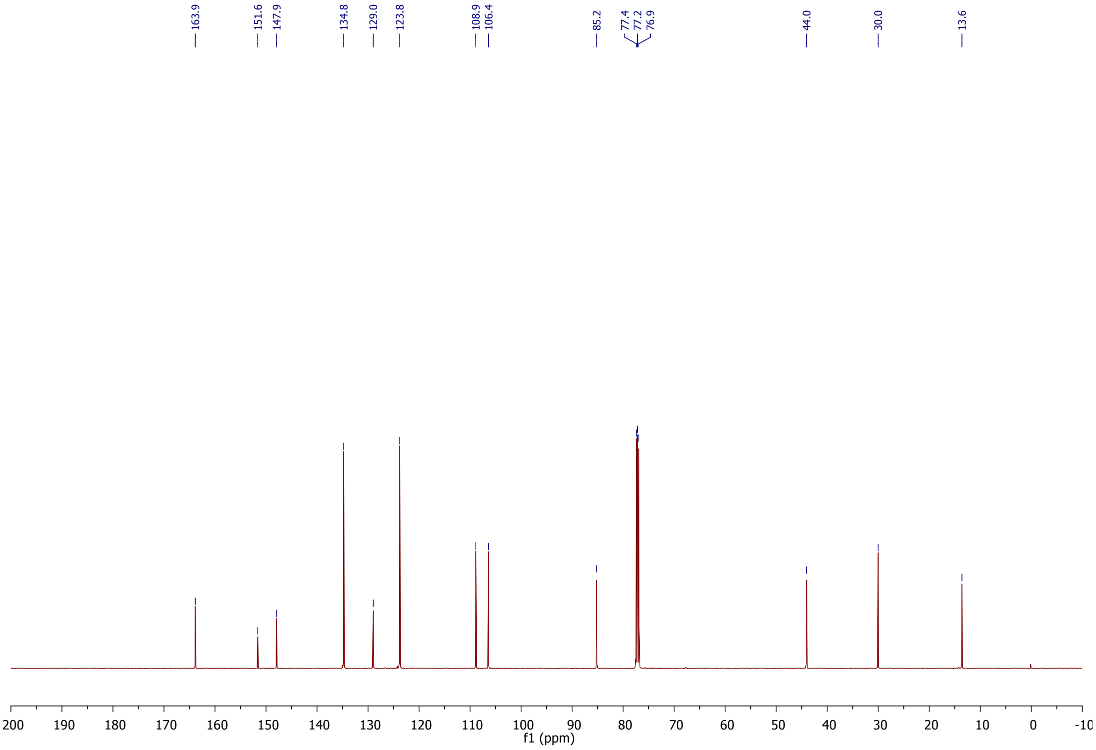

Figure S4. ^{13}C NMR spectrum of *(R)*-2-((1-chloro-3-(5-methylfuran-2-yl)propan-2-yl)oxy)isoindoline-1,3-dione (3).

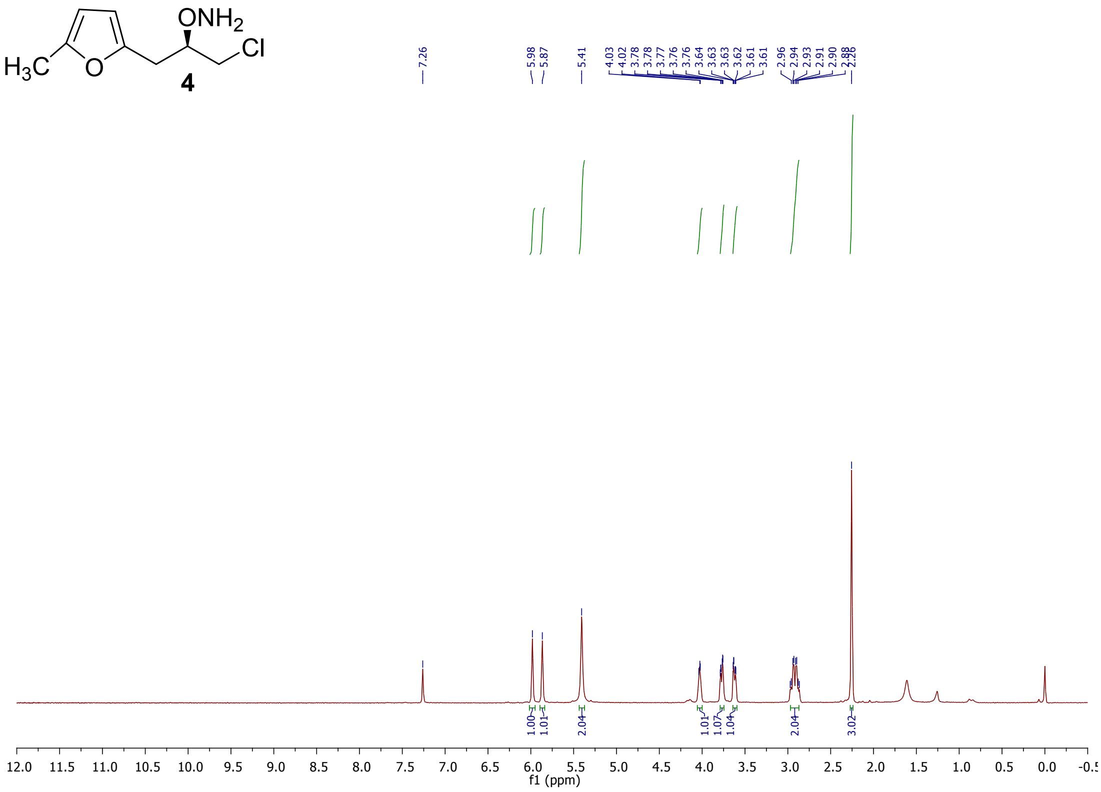

Figure S5. ^{1}H NMR spectrum of *(R)*-O-(1-chloro-3-(5-methylfuran-2-yl)propan-2-yl)hydroxylamine (4).

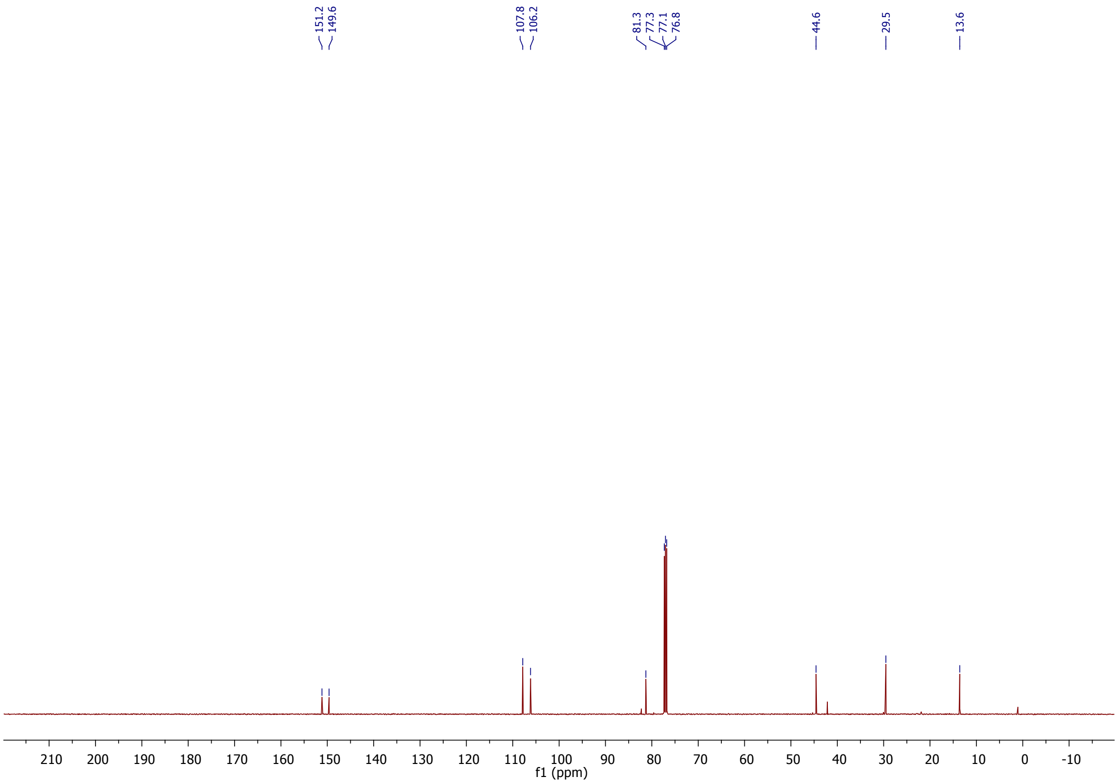

Figure S6. ^{13}C NMR spectrum of *(R)*-O-(1-chloro-3-(5-methylfuran-2-yl)propan-2-yl)hydroxylamine (4).

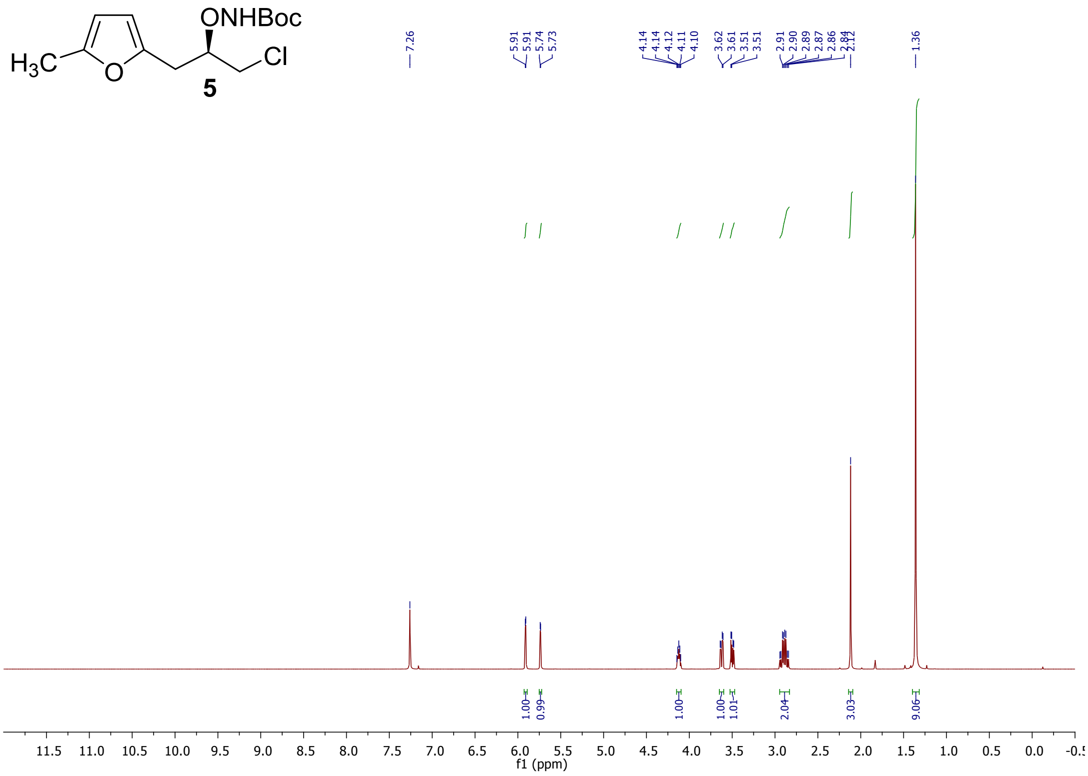

Figure S7. ^{1}H NMR spectrum of *tert*-butyl-*(R)*-((1-chloro-3-(5-methylfuran-2-yl)propan-2-yl)oxy)carbamate (5).

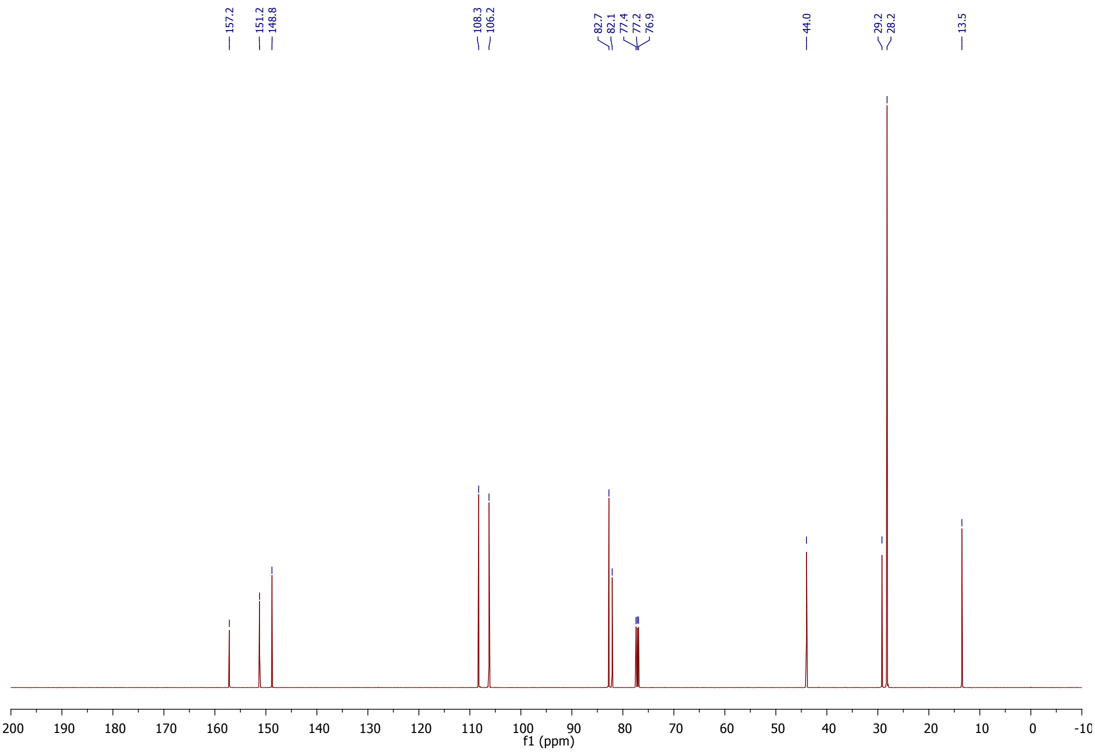

Figure S8. ^{13}C NMR spectrum of *tert*-butyl-*(R)*-((1-chloro-3-(5-methylfuran-2-yl)propan-2-yl)oxy)carbamate (5).

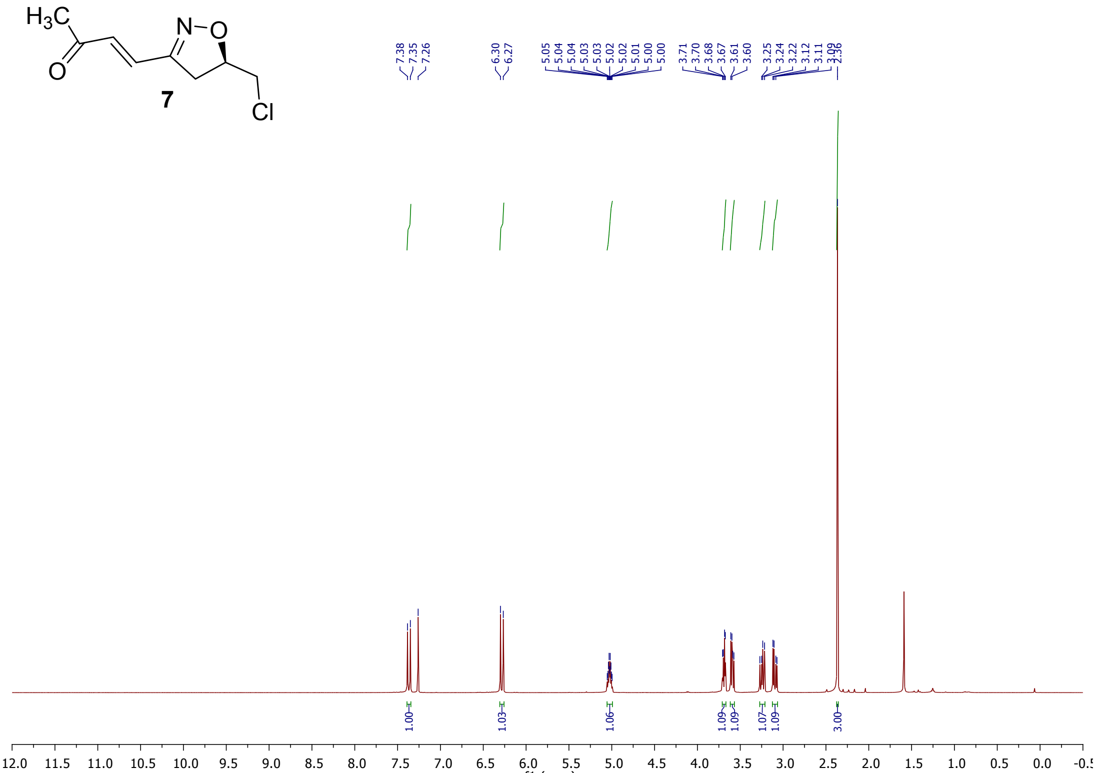

Figure S9. ^{1}H NMR spectrum of *(R,E)*-4-(5-(chloromethyl)-4,5-dihydroisoxazol-3-yl)but-3-en-2-one (7).

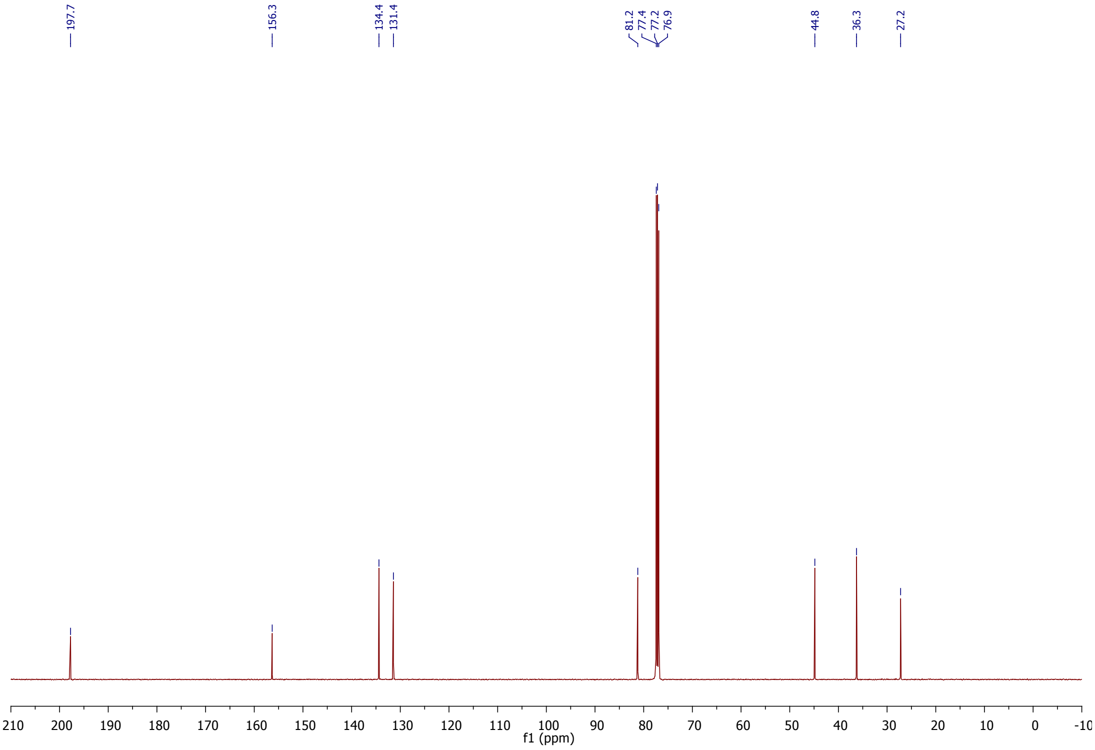

Figure S10. ^{13}C NMR spectrum of *(R,E)*-4-(5-(chloromethyl)-4,5-dihydroisoxazol-3-yl)but-3-en-2-one (7).

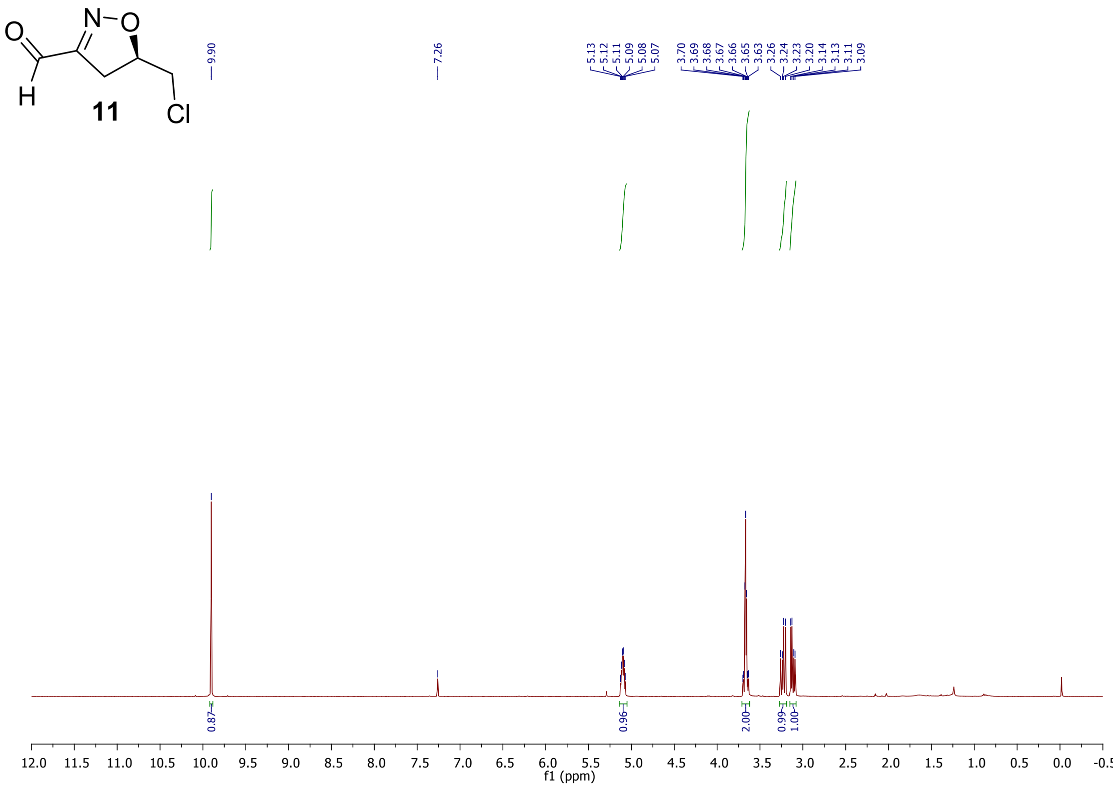

Figure S11. ^{1}H NMR spectrum of *(R)*-5-(chloromethyl)-4,5-dihydroisoxazole-3-carbaldehyde (11).

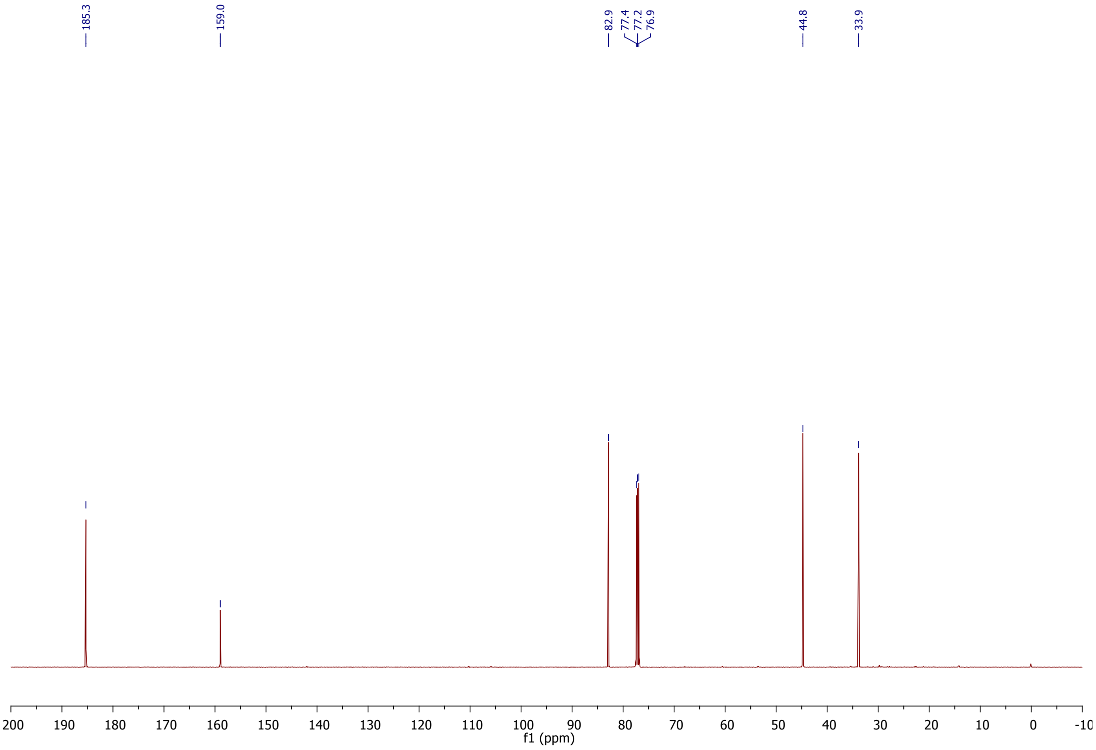

Figure S12. ^{13}C NMR spectrum of *(R)*-5-(chloromethyl)-4,5-dihydroisoxazole-3-carbaldehyde (11).

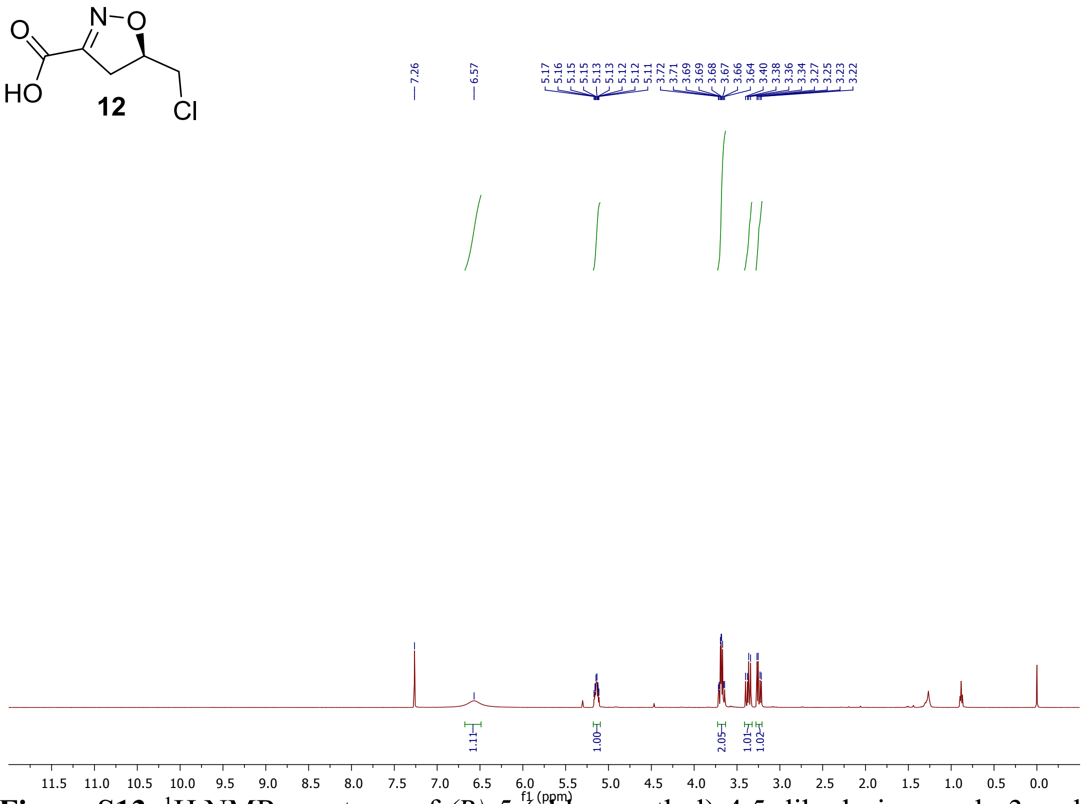

Figure S13. ^{1}H NMR spectrum of *(R)*-5-(chloromethyl)-4,5-dihydroisoxazole-3-carboxylate (12).

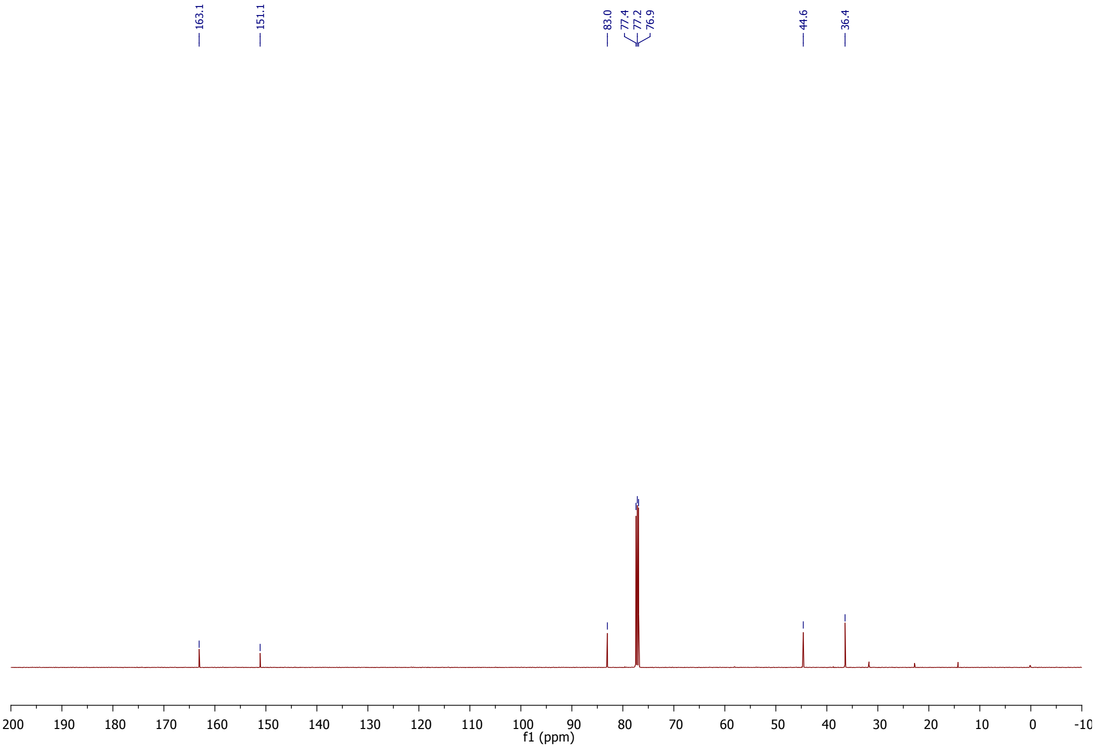

Figure S14. ^{13}C NMR spectrum of *(R)*-5-(chloromethyl)-4,5-dihydroisoxazole-3-carboxylate (12).

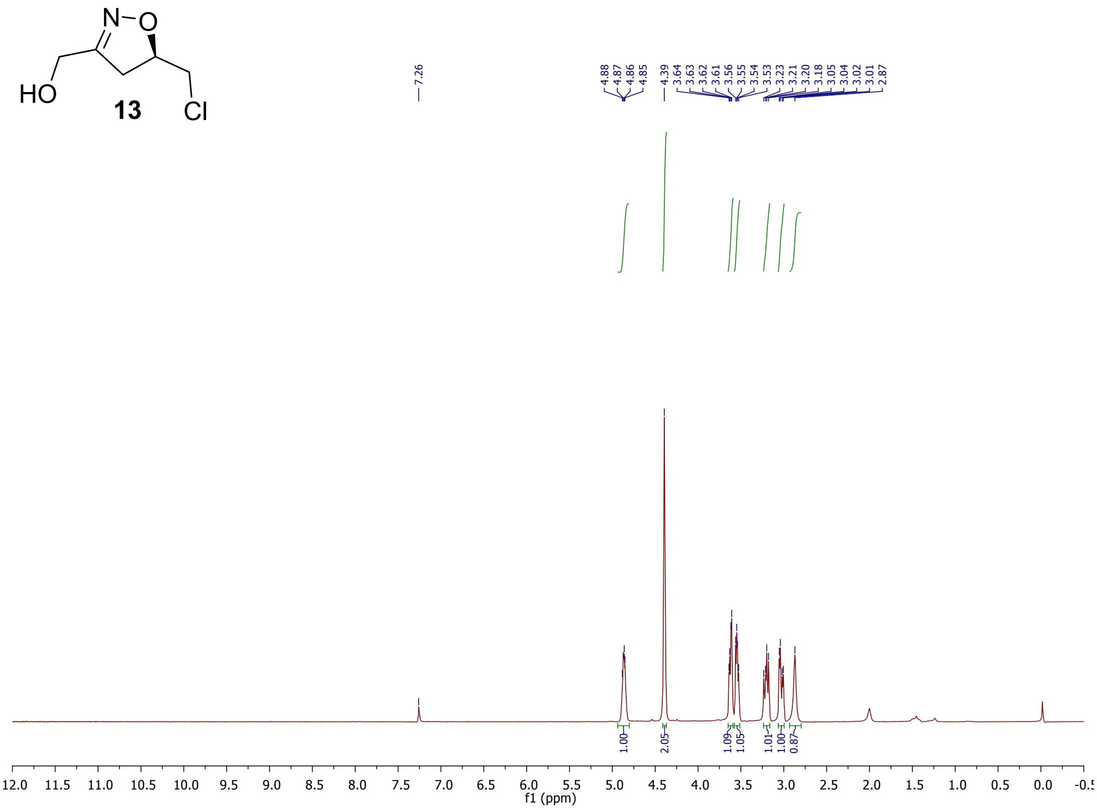

Figure S15. ^{1}H NMR spectrum of *(R)*-(5-(chloromethyl)-4,5-dihydroisoxazol-3-yl)methanol (13).

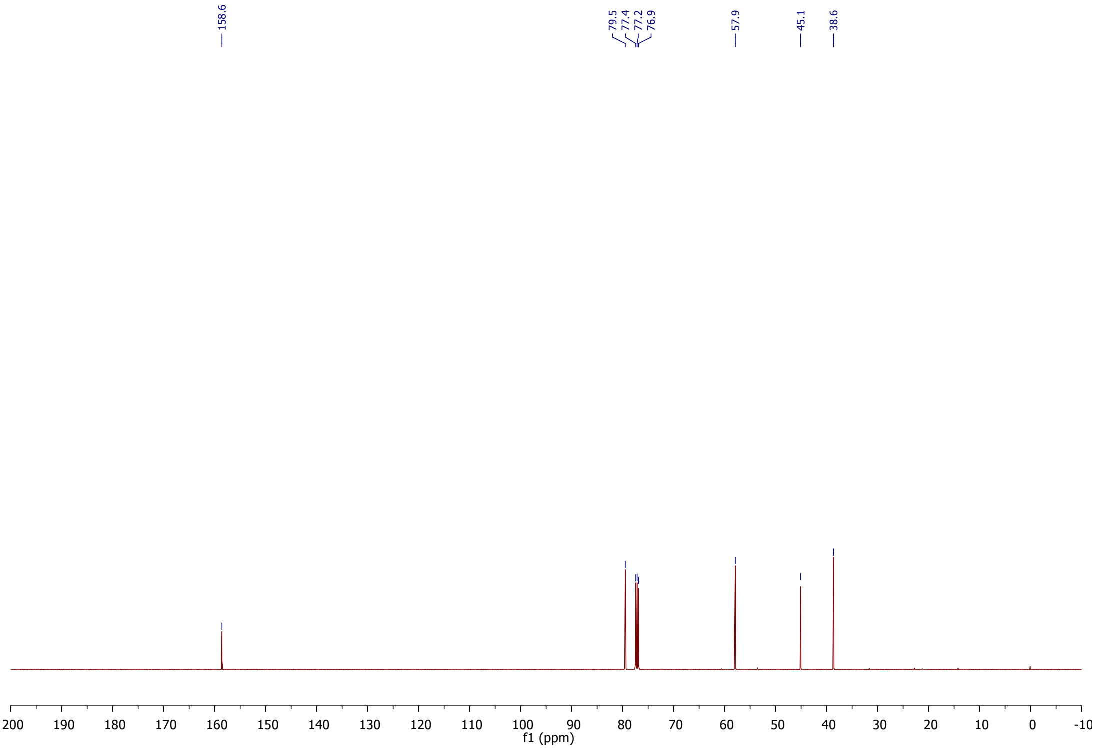

Figure S16. ^{13}C NMR spectrum of *(R)*-(5-(chloromethyl)-4,5-dihydroisoxazol-3-yl)methanol (13).

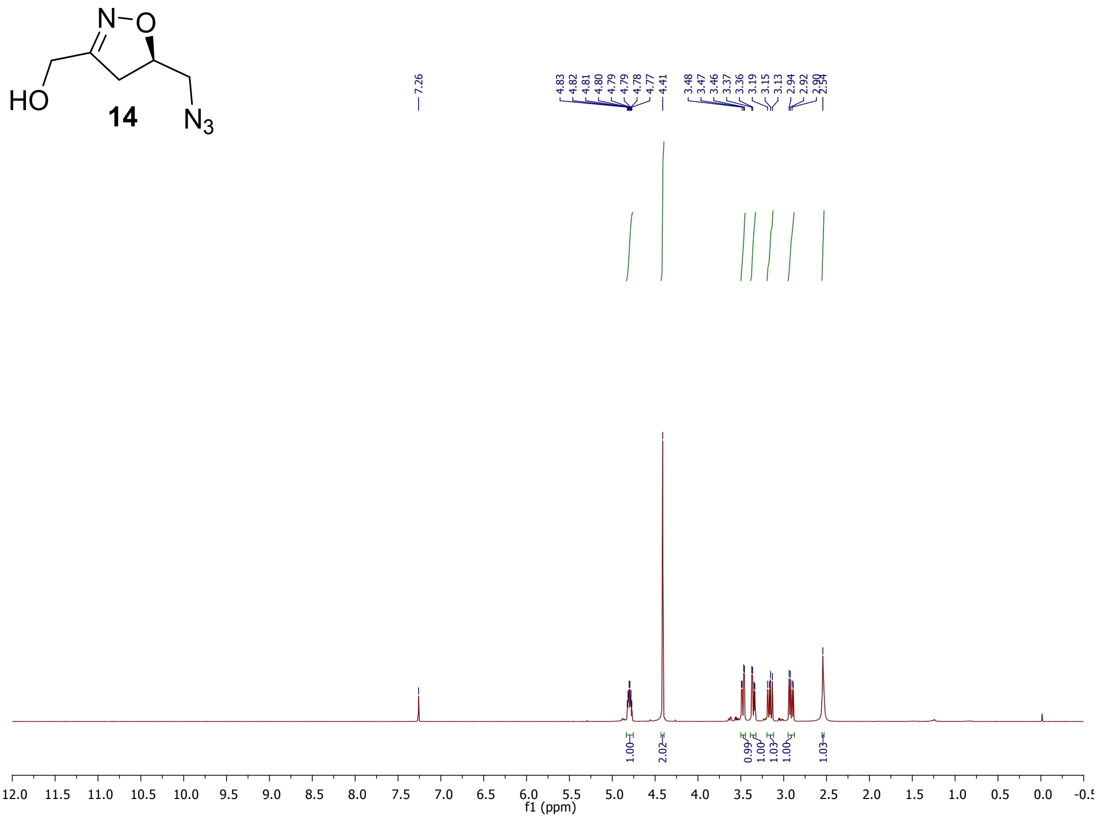

Figure S17. ^{1}H NMR spectrum of *(R)*-(5-(azidomethyl)-4,5-dihydroisoxazol-3-yl)methanol (14).

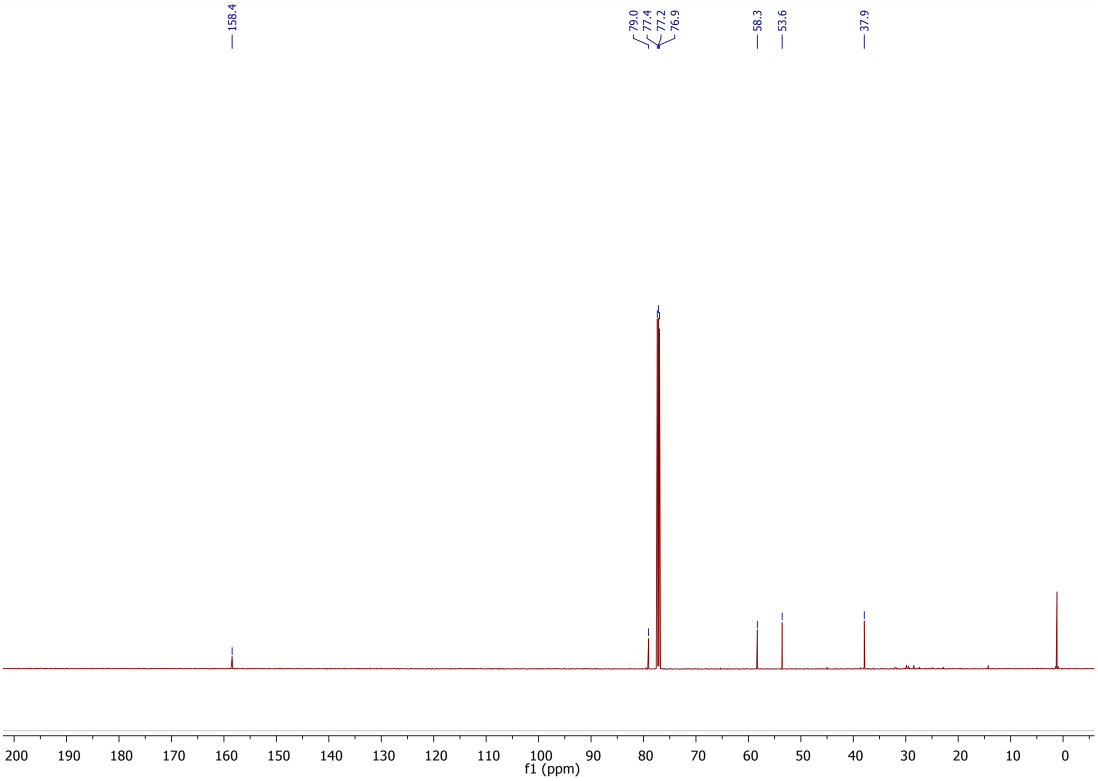

Figure S18. ^{13}C NMR spectrum of *(R)*-(5-(azidomethyl)-4,5-dihydroisoxazol-3-yl)methanol (14).

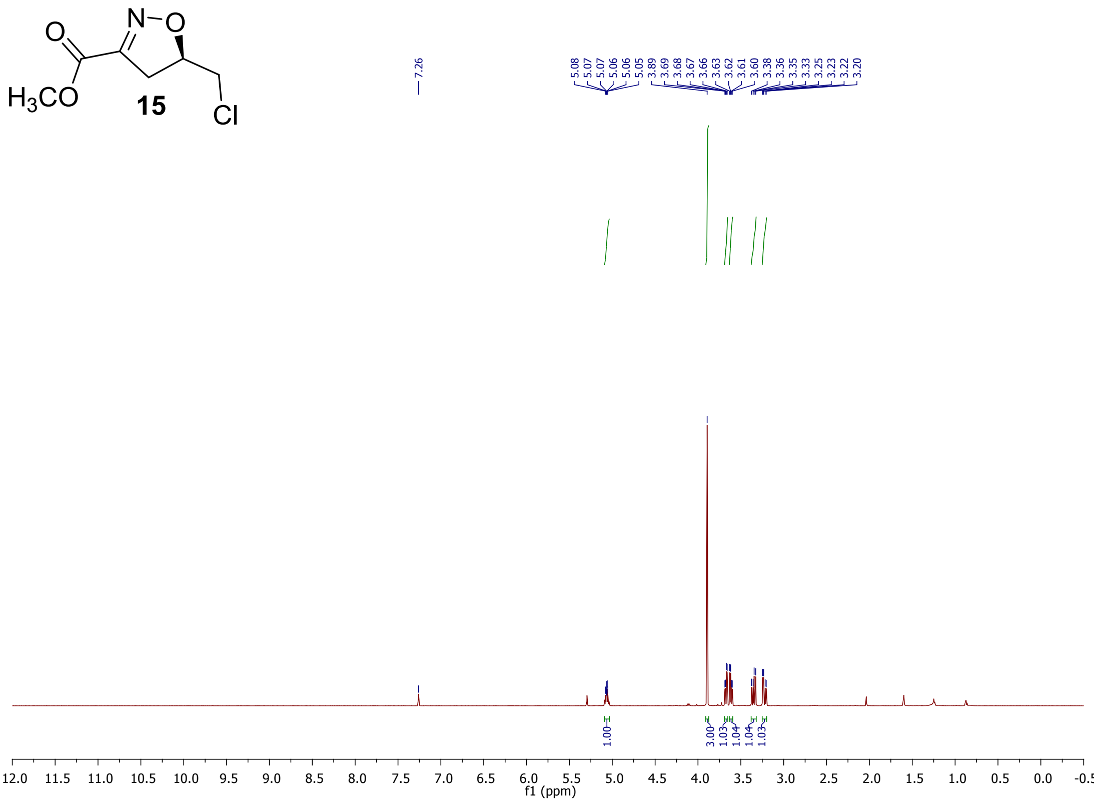

Figure S19. ^{1}H NMR spectrum of methyl *(R)*-5-(chloromethyl)-4,5-dihydroisoxazole-3-carboxylate (15).

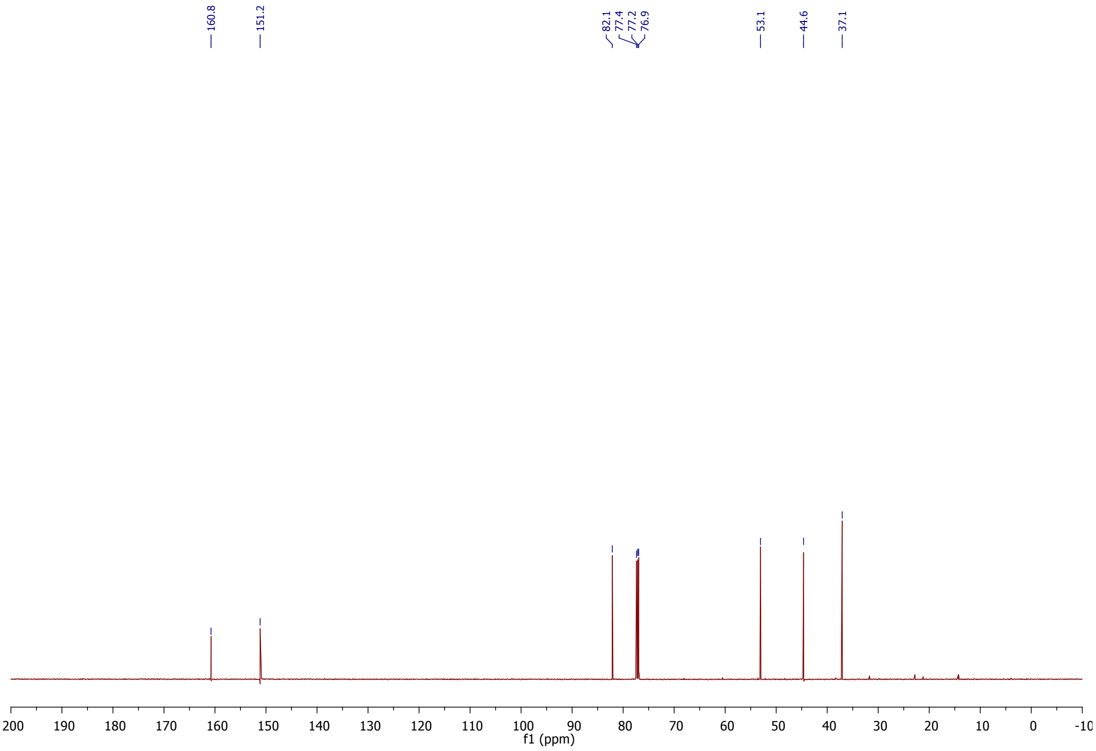

Figure S20. ^{13}C NMR spectrum of methyl *(R)*-5-(chloromethyl)-4,5-dihydroisoxazole-3-carboxylate (15).

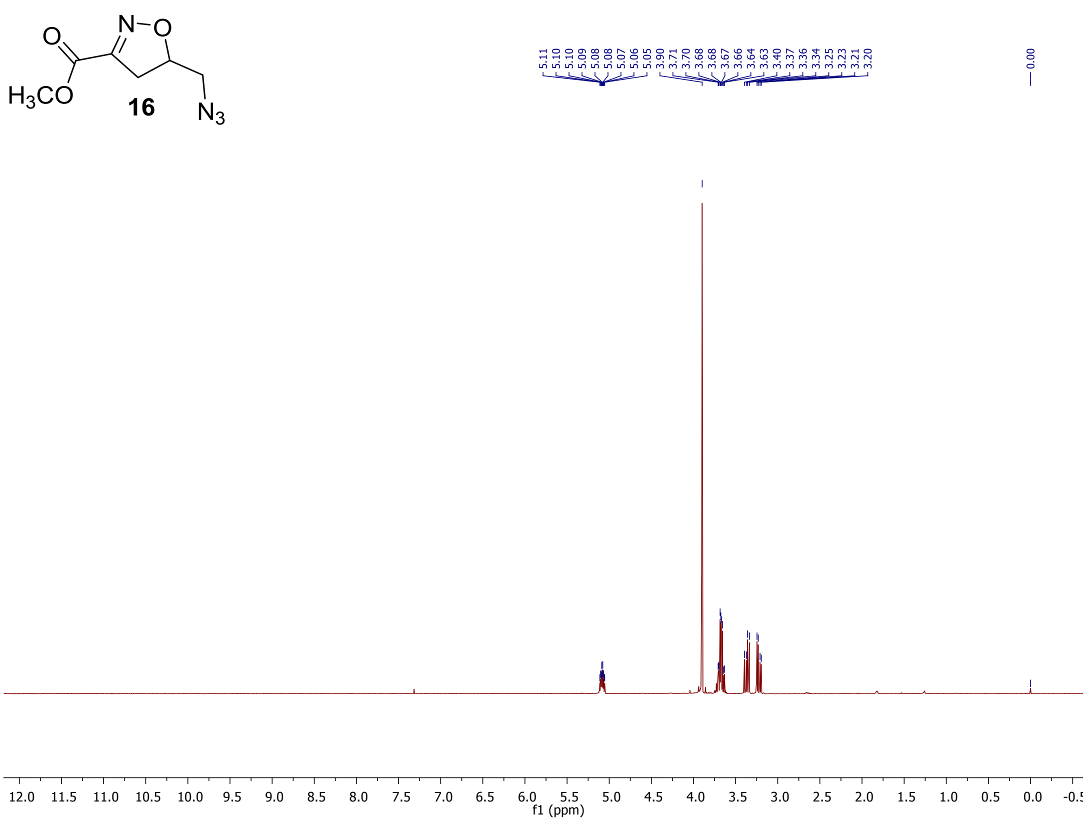

Figure S21. ^{1}H NMR spectrum of methyl *(R)*-5-(azidomethyl)-4,5-dihydroisoxazole-3-carboxylate (16).

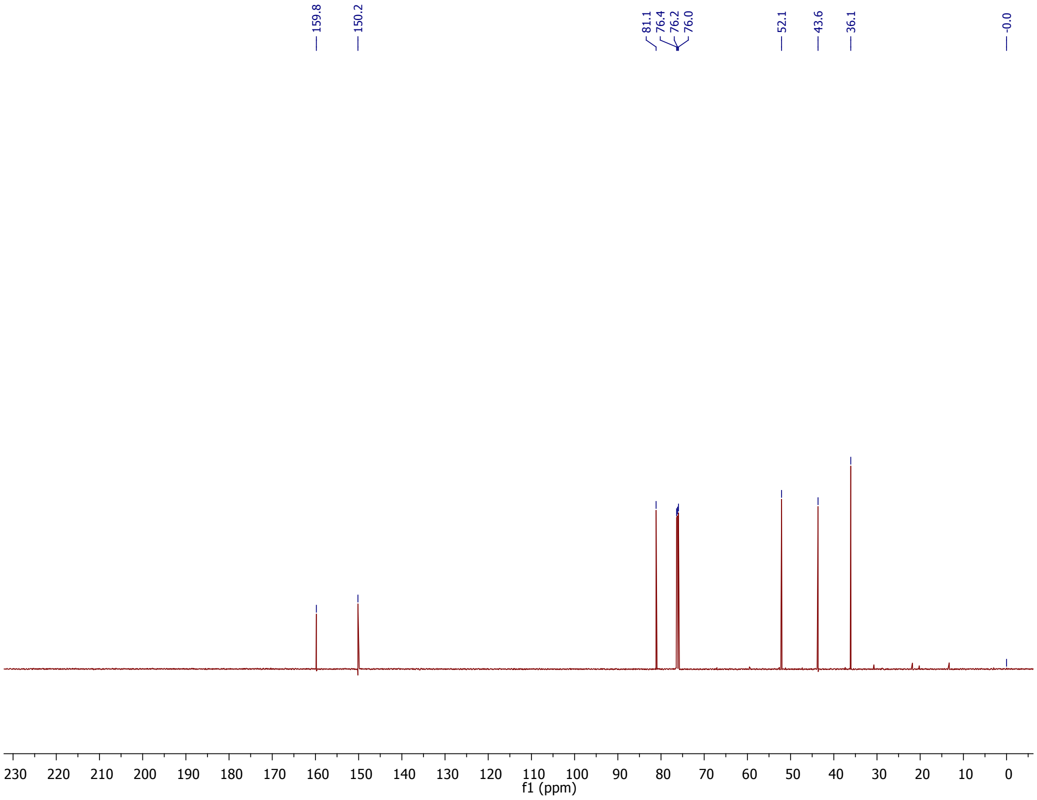

Figure S22. ^{13}C NMR spectrum of methyl *(R)*-5-(azidomethyl)-4,5-dihydroisoxazole-3-carboxylate (16).
